# Copy number variation in the human Y chromosome in the UK population

**DOI:** 10.1007/s00439-015-1562-5

**Published:** 2015-05-10

**Authors:** Wei Wei, Tomas Fitzgerald, Qasim Ayub, Andrea Massaia, Blair B. Smith, Anna A. Dominiczak, Andrew A. Morris, David D. Porteous, Matthew E. Hurles, Chris Tyler-Smith, Yali Xue

**Affiliations:** The Wellcome Trust Sanger Institute, Wellcome Trust Genome Campus, Hinxton, Cambridgeshire, CB10 1SA UK; School of Medicine, Ninewells Hospital and Medical School, Dundee University, Mackenzie Building, Kirsty Semple Way, Dundee, DD2 4RB UK; College of Medical, Veterinary and Life Sciences, University of Glasgow, Glasgow, G12 8QQ UK; School of Molecular, Genetic and Population Health Sciences, University of Edinburgh Medical School, Teviot Place, Edinburgh, EH8 9AG UK; Institute of Genetics and Molecular Medicine, Western General Hospital, University of Edinburgh, Crewe Road South, Edinburgh, EH4 2XU UK

## Abstract

We have assessed copy number variation (CNV) in the male-specific part of the human Y chromosome discovered by array comparative genomic hybridization (array-CGH) in 411 apparently healthy UK males, and validated the findings using SNP genotype intensity data available for 149 of them. After manual curation taking account of the complex duplicated structure of Y-chromosomal sequences, we discovered 22 curated CNV events considered validated or likely, mean 0.93 (range 0–4) per individual. 16 of these were novel. Curated CNV events ranged in size from <1 kb to >3 Mb, and in frequency from 1/411 to 107/411. Of the 24 protein-coding genes or gene families tested, nine showed CNV. These included a large duplication encompassing the *AMELY* and *TBL1Y* genes that probably has no phenotypic effect, partial deletions of the *TSPY* cluster and *AZFc* region that may influence spermatogenesis, and other variants with unknown functional implications, including abundant variation in the number of *RBMY* genes and/or pseudogenes, and a novel complex duplication of two segments overlapping the *AZFa* region and including the 3′ end of the *UTY* gene.

**Electronic supplementary material:**

The online version of this article (doi:10.1007/s00439-015-1562-5) contains supplementary material, which is available to authorized users.

## Introduction

Copy number variation (CNV) in the human genome contributes to both normal and pathological variation (Freeman et al. 2006). The Y chromosome is the most highly enriched of the human chromosomes for CNV in the general population (Redon et al. 2006), yet studies of Y-CNVs have lagged behind studies of the rest of the genome. For example, the high-resolution hybridization-based survey of Conrad et al. (2010) examined only females, while the sequence-based genomic surveys of the 1000 Genomes Project reported a total of five deletions on the Y, all smaller than 3 kb (Mills et al. 2011; The 1000 Genomes Project Consortium 2012). Similarly, recent surveys of medically relevant CNVs have been limited to specific studies focussed on a small number of known CNVs (Rozen et al. 2012).

This paucity of recent studies contrasts with early work in the field. Early cytogenetic studies revealed that the copy number of the entire Y chromosome in viable individuals could vary from zero (45,X; Turner Syndrome) to four (49,XYYYY) with only moderate phenotypic consequences (Paoloni-Giacobino and Lespinasse 2007; Legro 2012), while abundant variation in length of the Yq heterochromatin and the occasional presence of Nucleolar Organizer Regions (the cytogenetic manifestation of translocations of ribosomal DNA) were detected in surveys of the general population, and transmission observed in families (Jobling 2008). Molecular analyses using pulsed-field gel electrophoresis confirmed the high levels of variation in the heterochromatin in the general population, where detectable differences in the constituent tandemly repeated sequences DYZ1 and DYZ2 were universal, and often found between father and son pairs, and in addition discovered variation in the centromeric alphoid satellite DYZ3 and the tandemly repeated gene *TSPY* within the DYZ5 array (Oakey and Tyler-Smith 1990; Mathias et al. 1994). Two minisatellites (Jobling et al. 1998; Bao et al. 2000), abundant microsatellites (Kayser et al. 2004) and some retroposon insertions (Hammer 1994; Santos et al. 2000) have been reported. Molecular studies surveying the copy number of Y-specific loci similarly discovered general population duplications and deletions of segments of the chromosome that could be hundreds of kilobases or megabases in size (Jobling et al. 1996; Santos et al. 1998; Saxena et al. 2000; Bosch and Jobling 2003; Fernandes et al. 2004; Repping et al. 2004; Murphy et al. 2007; Balaresque et al. 2008, 2009). Rare pathological CNVs have also been identified, including cytogenetically visible deletions associated with spermatogenetic failure (Tiepolo and Zuffardi 1976) and anomalies of sex determination (Disteche et al. 1986) and three distinct cytogenetically undetectable deletions leading to spermatogenetic failure (Vogt et al. 1996), as well as insertions causing hearing impairment (Wang et al. 2013). In addition, CNVs with milder medically relevant effects have been identified: the gr/gr deletion in the *AZFc* region of Yq (Repping et al. 2003; Machev et al. 2004) and low *TSPY* copy number (Giachini et al. 2009), which both slightly increase the risk of spermatogenetic failure, while deletions that remove *AMELY* have no apparent phenotypic consequences, but confound DNA-based sex tests in forensic analyses (Santos et al. 1998). Thus, early work and later targeted analyses documented a rich variety of CNVs on the Y chromosome.

Subsequently, a genome-wide survey of CNVs using hybridization to BAC arrays revealed both that high levels of CNV associated with the *TSPY* array, centromere and *AZFc* region were readily detectable at this level of resolution in HapMap samples with African, European and East Asian ancestry, and also that detectable CNV outside these regions was infrequent in these samples (Redon et al. 2006). A targeted survey of some of the most frequent CNVs known by 2006 (*TSPY* array, *AZFc* region and Yq heterochromatin) confirmed the high levels of variation and high mutation rates at these loci in samples chosen to represent diverse branches of the Y-chromosomal phylogeny (Repping et al. 2006). However, as mentioned, our understanding of CNV on the Y chromosome has not benefited from more recent advances in array comparative genomic hybridization (array-CGH) resolution or whole-genome sequencing, and thus lags behind other chromosomes. Further CNV surveys including, or focusing on, the Y chromosome are needed.

We have performed the most comprehensive survey of Y-CNVs in the UK population thus far, discovering Y-CNVs using exome-focused array-CGH and validating them in a subset of samples using SNP-chip genotyping. We report here the rediscovery of several known Y-CNVs, the discovery of many novel ones, and their population-genetic and predicted functional properties.

## Materials and methods

### Subjects

We studied 411 unrelated apparently healthy UK males drawn from the UK Blood Service controls and the Scottish Family Health Study.

### Array-CGH

The array-CGH design, experimental procedures, QC, and CNV calling and merging have been described in detail elsewhere (The Deciphering Developmental Disorders Study 2015). Here, we briefly summarize the key features. The platform consisted of 2 × 1 M probe custom Agilent arrays (Amadid Nos. 031220/031221) with the probes targeted to (1) exons of protein-coding genes identified by GENCODE v17, with an average of two probes per exon and only 11 % of exons lacking probes, and (2) a genome-wide backbone with a median probe spacing of 5 kb. For chromosome Y, the platform contained a total of 6152 probes, covering 24 out of the 25 male-specific protein-coding genes/gene families (GENCODE v17). Probes in the X-transposed region and in the pseudoautosomal regions, which are not specific to the Y chromosome, were excluded from this analysis, so we did not call any CNVs from these regions. We were left with 5180 probes, of which 4974 (>96 %) are unique to the Y chromosome using the blastn program in the Blast + suite (http://blast.ncbi.nlm.nih.gov/Blast.cgi?CMD=Web&PAGE_TYPE=BlastDocs), with default parameters. So these probes are Y-specific by this criterion.

The reference sample used in all hybridizations was a pool of 500 males. CNVs were detected by CNsolidate with the default setting of a *w*-score threshold of 0.5 and the genome-wide false-positive (FPR) and false-negative (FNR) rates for CNsolidate raw calls were estimated by the DDD project (The Deciphering Developmental Disorders Study 2015). First, 73 technical replicates of the HapMap sample NA12878 were examined. True positives were defined as CNVs called in >80 % (i.e. 59) of the technical replicates, and CNVs were defined as the same if they shared greater than a 50 % reciprocal overlap. Overall, 12,634 true positives above the default *w*-score threshold of 0.5 were defined, 90 % of which (11,372) were classed as common and had been observed during previous studies at a population frequency >1 %. Using the default *w*-score threshold of 0.5 for CNV calls from CNsolidate resulted in a true-positive rate of 0.82 (FNR is <0.18), and a FPR of 0.052, across all replicates. Second, a custom designed 8 × 60 K Agilent CGH array was used to validate 9008 CNV calls, spanning the *w*-score range, detected by CNsolidate in 26 samples. Pearson correlation values of the mean log2 ratios across these samples between the discovery and validation arrays were used as the measure of truth. A clear 2-component distribution of correlation values was observed across all CNV calls. A nonparametric EM algorithm was used to determine the mixing proportions for each component and correlation values greater than the mean of the mixing proportions (0.5) were used to define true CNV calls. It showed that the proportion of true (validated) CNV calls was greater than 80 % for both losses and gains at the default 0.5 *w*-score cut-off for CNsolidate CNV calls.

Subsequently, additional manual curation was performed on the Y-CNVs to take account of the known repeated sequence content of the Y chromosome. Initially, rawCNVs in individual samples were merged into CNV events (CNVEs) when they overlapped, which we refer to as ‘rawCNVEs’. Then, since there is co-occurrence of some rawCNVEs in the same individual in this dataset, we merged some rawCNVEs into curated CNVEs ‘curCNVEs’. The logic was that if, for example, there are two copies of a related region in the reference sequence and most of the population, an individual with a deletion of one of these is expected to show a reduced signal at both locations, while an individual with a duplication will show an increased signal at both locations. The two locations will therefore show correlated signals in the population and do not represent independent events. We therefore examined the rawCNVEs for co-varying effects of this kind, and additional supporting evidence of sharing sequence homology. Several correlated signals were identified, and were noted to affect the *CDY*, *DAZ*, *PRY*, *RBMY* and *TSPY* genes; we discuss specific examples in the “Results and discussion” section. In two cases each found in a single individual, two unique rawCNVEs specific to that individual lay close together on the chromosome. The combination of occurrence of the rawCNVEs together in one individual and absence from all other individuals, together with physical proximity, suggested that they were likely to result from a single complex mutational event. These examples (curCNVE8 and curCNVE14) are also discussed further in the “Results and discussion”. Thus, taking all these factors into account, a set of curated CNVEs (curCNVEs) was created and their sizes were refined by manual inspection of probe intensity plots.

### SNP-chip genotyping

The SNP genotyping platform and procedures have also been described in detail elsewhere (The Deciphering Developmental Disorders Study 2015). In brief, a customized Illumina Omni-one quad chip was used containing 811,844 mapped and 1734 unmapped markers, with a median intermarker distance of 2378 bp (SangerDDD_OmniExPlusv1_15019773_A). For chromosome Y, the platform contained a total of 1681 probes, covering 12 out of 25 protein-coding genes/gene families (GENCODE v17). Log *R* ratios (LRR) were used in the current study. Data were available for 149 of the 411 individuals.

### Y haplogroup assignment

Y haplogroups were identified from allele calls at standard diagnostic Y-SNPs (Karafet et al. 2008) that were present on the SNP-chip array, as well as other informative markers (Fig. 4, Figure S2; Table S2), and were thus available for 149 individuals. Haplogroups were considered at a maximum phylogenetic resolution of the trinomial level, e.g. R1b. A full Y phylogeny as defined by all markers typed was constructed (Figure S2).

## Results and discussion

### CNV calling, validation and curation

Y-chromosomal array-CGH data were analysed from 411 UK males, representative of the general UK population. RawCNVs were called by CNsolidate and combined into rawCNVEs as described in the "Materials and methods" section. In addition, SNP genotype data were available for 149 of the participants, and so when a SNP overlapped with a rawCNVE call, the SNP intensity data could be used to assess whether or not there was independent support for the CNVE call.

**Fig. 1:**
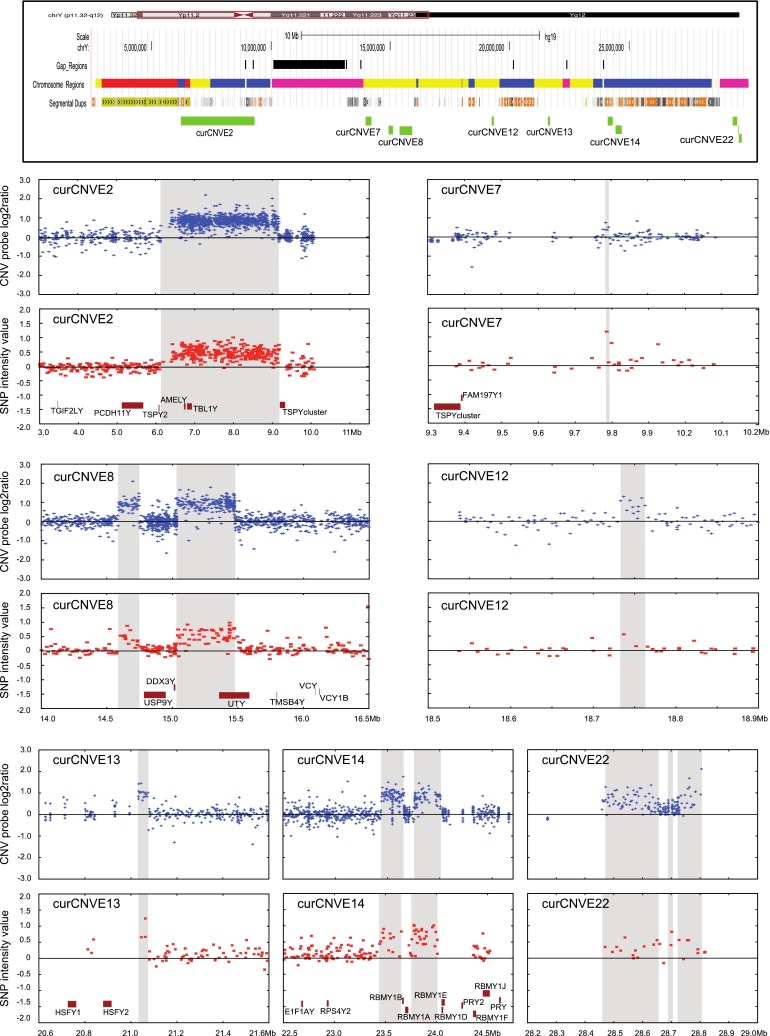
Validated examples of CNVs based on both CNV probe and SNP intensity data. *Top panel* male-specific euchromatic region of the Y chromosome derived from the UCSC genome browser showing gaps in the reference sequence (*black bars*), chromosomal regions (*yellow* Y-specific unique, *red* X–Y transposed, *blue* Y-specific repeated, *purple* heterochromatic), segmental duplications and the CNVEs illustrated in the rest of the figure. *Remaining panels* paired probe intensity (*blue*) and SNP intensity (*red*) plots for seven curCNVEs. Each rawCNVE is indicated by *buff shading*, chromosomal coordinates in Mb are shown at the *bottom* and overlapping protein-coding genes within the plot regions are included below the SNP intensities

We took a three-stage approach to evaluating the starting rawCNVE calls. First, we used the comparison of array-CGH probe intensity with SNP intensity, together with manual examination of the two datasets by two independent individuals, to identify a set of validated rawCNVEs, where the array-CGH calls were supported by the SNP data. The two most ‘borderline’ examples, which illustrate the procedure, were curCNVE7 and curCNVE12 (Fig. 1). curCNVE7 was considered validated because it had strong support from two SNPs, which showed the highest SNP intensity values in the 1 Mb window illustrated. For curCNVE12, where the array-CGH signal was very strong, a single SNP showing the highest SNP intensity value in the 400 kb window illustrated was considered sufficient to validate it. Second, we applied the array-CGH probe intensity criteria established in the first stage to the remaining rawCNVE calls, which did not have SNP intensity data, again with manual examination, to identify a set of likely rawCNVEs. Third, we used literature data to determine whether or not the validated and likely rawCNVEs were supported by previous work, and also whether known common CNVs were being called.

**Fig. 2:**
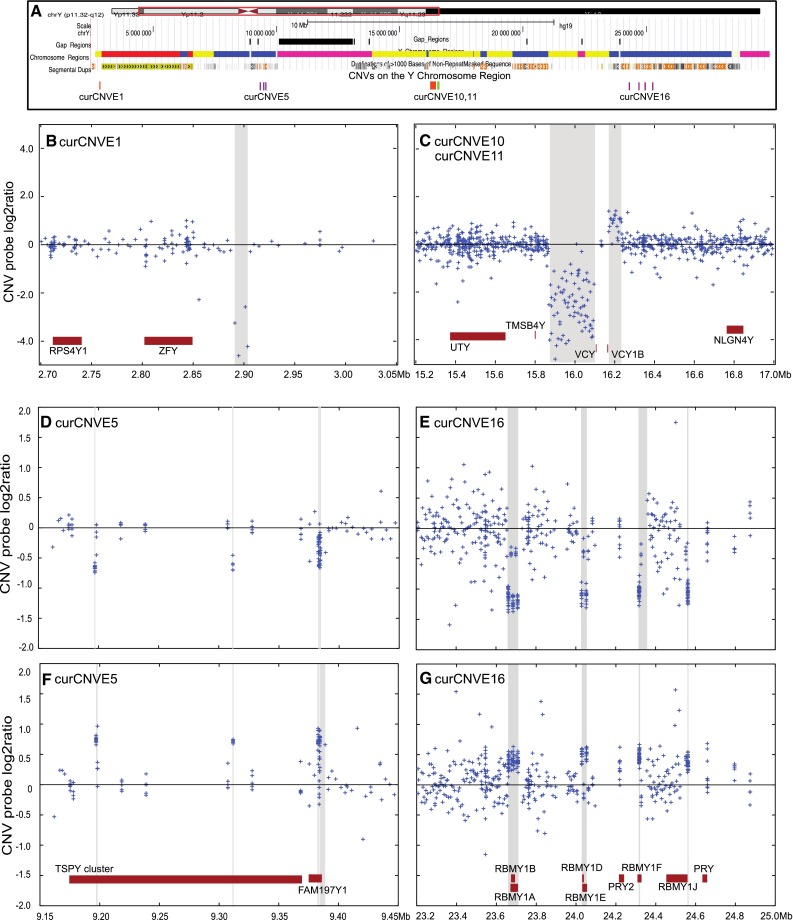
Likely examples of CNVs based on CNV probe intensity data. **a** Male-specific euchromatic region of the Y chromosome derived from the UCSC genome browser showing gaps in the reference sequence (*black bars*), chromosomal regions (*yellow* Y-specific unique, *red* X–Y transposed, *blue* Y-specific repeated, *purple* heterochromatic), segmental duplications and the CNVEs illustrated in the rest of the figure (*green* duplications, *orange* deletions, *purple* both). **b** curCNVE1. **c** curCNVEs 10 and 11. **d** and **f** curCNVE5 in two different individuals showing the coordinated decrease or increase of rawCNVEs 5.1, 5.2 and 5.3. **e** and **g** similar plots for curCNVE16 and its corresponding rawCNVEs. Each rawCNVE is indicated by *buff shading*, chromosomal coordinates in Mb are shown at the *bottom* and overlapping protein-coding genes are plotted at the *bottom* of **b** and **c**, **f** and **g**

Manual curation of the rawCNVE calls in the 149 individuals with both array-CGH and SNP data identified a set of calls with strong evidence for variation in copy number, and examples of these validated rawCNVEs are shown in Fig. 1. All are >7 kb in size, as expected from the requirement to contain both multiple array and SNP probes. Similar curation using the array probe intensity data alone in these and the remaining 262 individuals identified additional CNVs in regions that did not overlap with SNPs, or in the individuals without SNP data. Examples of these likely rawCNVEs are shown in Fig. 2. They included some smaller CNVEs, such as rawCNVE5.2 where three probes lay within 165 bp (Fig. 2d, f). During curation, we combined some of the rawCNVEs into curCNVEs. In deciding whether or not rawCNVEs should be combined, we considered correlations between probe intensity signals in different individuals to determine whether multiple individual rawCNVEs co-vary in the population: for example, when one rawCNVE shows a log2 ratio increase in a particular individual, do other rawCNVEs show this pattern as well; and similarly when one shows a decrease? If they did this consistently, they were combined in the same curCNVE. Additional supporting information taken into account was whether or not all co-varying CNVs were known to share sequence homology (as for the shared RBMY elements of curCNVE16, Fig. 2) or if the co-varying CNVs lay close together on the chromosome, so that a single mutational event could plausibly have affected them all. For example, rawCNVEs 5.1, 5.2 and 5.3 all showed decreased signal indicating a decrease in copy number in one individual (Fig. 2d), and increased signal and copy number in a different individual (Fig. 2f). A similar pattern was seen for rawCNVEs 16.1, 16.2, 16.3 and 16.4 (Fig. 2e, g). These rawCNVEs are therefore each likely to represent a single event where probes cross-hybridize, as the (moderately) repeated regions show the copy number change, while the unique regions do not. We therefore conclude that in cases like these, a single copy number change in a *TSPY*-related sequence (curCNVE5) or an *RBMY* gene (curCNVE16) could generate the observed signal because of cross-hybridization.

In addition, some rawCNVEs were detected in single individuals, lay close together in the genome and showed similar changes: rawCNVE8.1 and rawCNVE8.2 (Fig. 1), and rawCNVE14.1 and 14.2 (Fig. 1). The two distinct rawCNVEs in both cases behaved in the same way in the population (duplication one individual, no change in the rest of the individuals investigated); also, in both cases, the two regions lay within 1 Mb on the chromosome. In cases like the four discussed, we considered the rawCNVEs as likely to reflect the same mutational event, and in a second round of curation grouped them together as curCNVEs: 5, 16, 8 and 14, respectively. When nearby rawCNVEs in the same single individual showed contrasting signals, such as the deletion at rawCNVE10 and duplication at rawCNVE11 (Fig. 2c), we did not group them, and retained them as curCNVE10 and curCNVE11, respectively.

381 raw Y-specific CNVE calls from 185 individuals were accepted at 34 rawCNVE loci, an average of 0.93 per individual (range 0–4). No rawCNVE was accepted in 226 individuals: 36 had no raw calls at all on the Y, 10 had raw calls only outside the MSY region, and 180 individuals with raw calls in MSY region did not pass the manual check. Overall, the raw calls were consolidated into 22 curCNVE loci. A single curCNVE could include both duplications and deletions of a particular region. The full call set is shown in Table S1 and examples of each are illustrated in Supplementary Fig. 1.

### General characteristics of validated and likely CNVEs

**Fig. 3:**
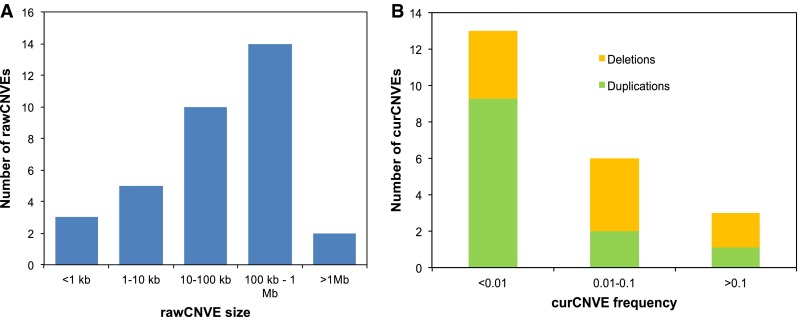
Size and frequency distribution of validated and likely CNVs in 238 individuals. **a** rawCNVE size distribution. **b** curCNVE frequency distribution; each frequency *bar* is coloured according to the proportion of duplications (*green*) and deletions (*orange*) among the total 381 curCNVE calls

The sizes of rawCNVEs ranged from <1 kb to >3 Mb [Table 1; Fig. 3a; the mean size was 309 kb (median 72 kb)]. These large sizes reflect the low probe and SNP densities, and the need for a signal at multiple probes/SNPs to make confident calls; since curCNVEs can be discontinuous, their summed sizes are not easily interpreted and we do not consider them. Frequencies ranged from 1/411 (0.24 %) to 107/411 (26.0 %) (Fig. 3b). More than half (12/22, ~55 %) were observed in just one individual, but six were called in more than 5 % (Fig. 3b). Among the 381 curCNVE calls across all samples, deletions (240) outnumbered duplications (141), a statistic dominated by the 76 deletions at curCNVE16 and 68 deletions at curCNVE5 (Table 1). Six of the curated CNVEs have been reported previously and the remaining 16 are novel.

**Table 1:** Summary of 34 rawCNVEs and 22 curCNVEs called in this study

curCNVE	rawCNVE	rawCNVE				curCNVE				
		Start	End	Size (bp)	SNP support	Known	Duplications	Deletions	Total frequency	Protein-coding gene content^a^
curCNVE1	rawCNVE1	2,891,036	2,903,671	12,635				1	0.0024	
curCNVE2	rawCNVE2	6,138,072	9,161,980	3,023,908	+	Murphy et al. (2007)	1		0.0024	*AMELY, TBL1Y*
curCNVE3	rawCNVE3	7,659,321	8,497,398	838,077			1		0.0024	
curCNVE4	rawCNVE4	9,170,730	9,175,364	4634			53	11	0.1557	*TSPY4*
curCNVE5	rawCNVE5.1	9,196,977	9,198,235	1258			8	68	0.1849	*TSPY8*
curCNVE5	rawCNVE5.2	9,311,600	9,311,765	165						
curCNVE5	rawCNVE5.3	9,383,079	9,384,475	1396						
curCNVE6	rawCNVE6	9,196,977	9,382,943	185,966		Oakey and Tyler-Smith (1990)	2	29	0.0754	*TSPY cluster*
curCNVE7	rawCNVE7	9,785,127	9,792,677	7550	+		1		0.0024	
curCNVE8	rawCNVE8.1	14,588,389	14,745,226	156,837	+		1		0.0024	
curCNVE8	rawCNVE8.2	15,034,145	15,475,430	441,285	+					*UTY*
curCNVE9	rawCNVE9	15,144,435	15,146,222	1787				1	0.0024	
curCNVE10	rawCNVE10	15,869,445	16,096,260	226,815				1	0.0024	
curCNVE11	rawCNVE11	16,170,165	16,233,113	62,948			1		0.0024	
curCNVE12	rawCNVE12	18,733,053	18,762,614	29,561	+		1		0.0024	
curCNVE13	rawCNVE13	21,032,549	21,074,621	42,072	+		1		0.0024	
curCNVE14	rawCNVE14.1	23,441,081	23,649,415	208,334	+		1		0.0024	
curCNVE14	rawCNVE14.2	23,756,420	24,005,801	249,381	+					
curCNVE15	rawCNVE15	24,218,723	24,218,783	60			18	1	0.0462	*PRY2*
curCNVE16	rawCNVE16.1	23,660,808	23,709,077	48,269			31	76	0.2603	*RBMY1B, RBMY1A1*
curCNVE16	rawCNVE16.2	24,005,497	24,062,091	56,594						*RBMY1D, RBMY1E*
curCNVE16	rawCNVE16.3	24,316,281	24,327,019	10,738						*RBMY1F*
curCNVE16	rawCNVE16.4	24,551,695	24,562,435	10,740						*RBMY1J*
curCNVE17	rawCNVE17.1	24,551,695	24,658,825	107,130			4	19	0.0560	*RBMY1J, PRY*
curCNVE17	rawCNVE17.2	24,551,695	24,795,554	243,859						*RBMY1J, PRY*
curCNVE18	rawCNVE18	25,130,433	27,895,495	2,765,062		Fernandes et al. (2004)	8	17	0.0608	*BPY2, DAZ1, DAZ2, CDY1B, BPY2B, DAZ3, DAZ4, BPY2C, CDY1*
curCNVE19	rawCNVE19	24,658,743	25,428,575	769,832		Repping et al. (2003)	2	9	0.0268	*BPY2, DAZ1, DAZ2*
curCNVE20	rawCNVE20.1	25,284,428	25,428,580	144,152		Saxena et al. (2000)	7	6	0.0316	*DAZ1, DAZ2*
curCNVE20	rawCNVE20.2	26,950,819	27,177,168	226,349						*DAZ3, DAZ4*
curCNVE21	rawCNVE21.1	25,829,578	26,194,226	364,648		Machev et al. (2004)	1	1	0.0049	*CDY1B*
curCNVE21	rawCNVE21.2	27,768,203	27,768,295	92						*CDY1*
curCNVE22	rawCNVE22.1	28,472,070	28,654,473	182,403	+		1		0.0024	
curCNVE22	rawCNVE22.2	28,688,829	28,704,081	15,252	+					
curCNVE22	rawCNVE22.3	28,723,589	28,804,541	80,952	+					

Genome coordinates are based on GRCh37/hg19. Gene names are from GENCODE v20

^a^Genes showing CNV. For genes that are members of families, the copy that is actually duplicated or deleted is unknown because of shadowing effects

### Distribution of curCNVEs among Y haplogroups

**Fig. 4:**
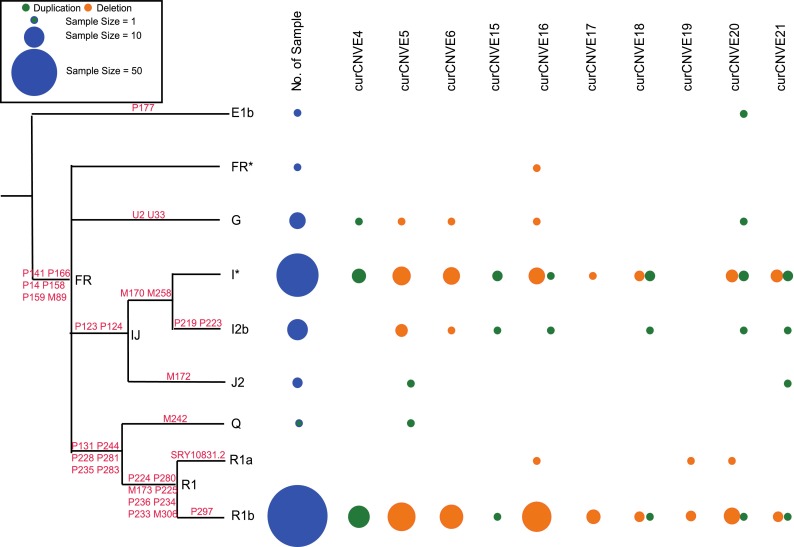
Haplogroup distribution of curCNVEs present in more than one individual. *Left* phylogeny of the Y-chromosomal haplogroups detected in 149 samples; branch lengths are arbitrary. *Blue circles* haplogroup frequency in the entire 149 individuals, with circle area proportional to frequency. *Remaining circles* haplogroup frequencies in individual curCNVEs

The absence of recombination in the male-specific portion of the Y chromosome results in a simple phylogenetic tree that can be defined by Y-SNPs (Jobling and Tyler-Smith 2003; Wei et al. 2013). With the SNPs available in this study, we could assign all 149 samples with SNP genotype data to trinomial level haplogroups, and the haplogroup distribution is as expected in the UK population (Fig. 4; Table S1; Figure S2; Table S2) (Capelli et al. 2003). curCNVEs can then be placed on the known tree, and a minimal number of mutational events leading to each curCNVE can be deduced. curCNVEs confined to a single haplogroup, or cluster of phylogenetically related haplogroups, can be most parsimoniously explained by a single mutational event, while curCNVEs dispersed among unrelated haplogroups require multiple mutational events.

Applying this reasoning to the 10 curCNVEs present in more than one individual, involving 62 of the 149 samples (Fig. 4), shows that all require multiple mutations to explain their phylogenetic distribution, a conclusion reinforced by the observation that both duplications and deletions were called at all of these 10 loci (Table 1), although haplogroup assignments were not available in all cases (Fig. 4).

### Biological impact of curCNVEs

The Y chromosome codes for 25 male-specific proteins, and 24 of these are covered by probes on the CGH array. The 22 curCNVEs together overlap with genes that code for nine of these proteins (AMELY, TBL1Y, TSPY, UTY, CDY, RBMY, PRY, BPY2 and DAZ), with the caveat that due to shadowing effects (where there are multiple copies of these genes or pseudogenes in the reference sequence), we cannot always be sure whether the copy number variation affects the functional gene(s) or non-functional pseudogenes(s).

Since duplication and deletion of the *AMELY/TBL1Y* (Santos et al. 1998; Murphy et al. 2007), *TSPY* (Oakey and Tyler-Smith 1990; Mathias et al. 1994; Repping et al. 2006) and the Yq region containing the *CDY, BPY2 and DAZ* genes (Jobling et al. 1996; Repping et al. 2003, 2004, 2006; Fernandes et al. 2004) have been extensively documented in the literature, and can have subtle biological consequences (Repping et al. 2003; Machev et al. 2004; Giachini et al. 2009), we focus here on the remaining *UTY*, *RBMY* and *PRY* genes.

**Fig. 5:**
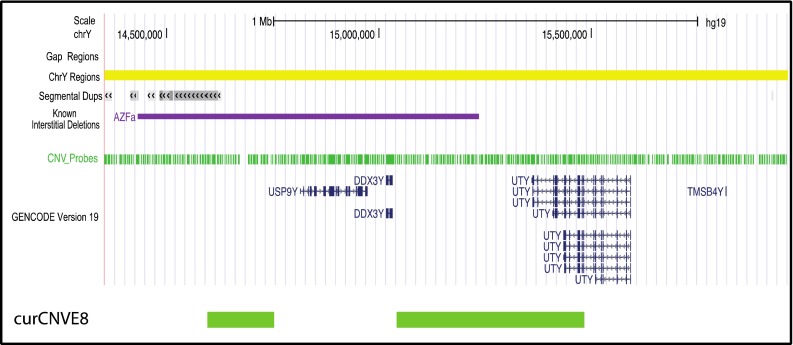
Novel partial duplication of *UTY*. curCNVE8 showing the relationship with the *AZFa* deletion and the protein-coding genes in the region

Deletion of a region of the Y chromosome between 14,434,311 and 15,228,218 carrying the *USP9Y* and *DDX3Y* genes (the *AZFa* region) leads to azoospermia (Tyler-Smith and Krausz 2009), but duplication of the same region is present in the general population and compatible with male fertility (Bosch and Jobling 2003). Partial *AZFa* deletions have consequences that range from azoospermia to normozoospermia, but partial duplications have not previously been reported. curCNVE8, found in a single individual, consists of duplications of two discontinuous regions overlapping with *AZFa* although these do not include *USP9Y* or *DDX3Y*. curCNVE8 does, however, extend beyond the distal boundary of *AZFa* and duplicate the 3′ end of the *UTY* gene (Fig. 5). UTY is a histone demethylase (Walport et al. 2014) but this partially duplicated copy seems unlikely to be expressed and should be considered a variant of unknown significance.

*RBMY* and *PRY* both form multicopy gene families on the Y chromosome containing pseudogenes as well as six and two active genes, respectively. curCNVEs 14–17 include members of these families, and curCNVE16 containing *RBMY* genes (Fig. 2e, g) is the most common CNV detected (Table 1). However, because the CNV analysis does not distinguish between genes and pseudogenes, and there is an *RBMY* pseudogene located at 9,148,467–9,162,451 (which contains only a single probe and thus does not permit reliable CNV measurement), the biological implication of the variation detected remains uncertain.

The DDD project has carried out false-positive and false-negative rate assessments using both technical replicates and custom designed array validation (see “Materials and methods”), which showed that the FNR is <20 % and FPR is about 5 %. However, these measurements have only limited application to our study, as we did not use the automatic calls in their raw state as our final call set. Instead, we manually examined all the rawCNV calls one by one, a procedure which we regard as gold standard. We also took into account the complication of repeated Y chromosome structures, and the evidence for the 22 curCNVEs we accepted was compelling. So the false-positive rate among our curCNVEs is likely to be even lower (Figs. 1, 2), perhaps zero. In contrast, the false-negative rate is unknown but likely to be high. This is an inevitable consequence of the limited probe coverage. Mills et al. (2011) showed that genome-wide numbers of CNVs increase as size decreases, at least down to the 100 bp resolution limit of their analysis. Since we have essentially no power to discover CNVs of 100 bp, we must be missing a lot of small ones from our call set. We also potentially miss CNVs in sequences present on some Y chromosomes but absent from haplogroup R1b, since the array-CGH probes were designed based on the reference sequence, which is mostly derived from an R1b chromosome. Even with the current ‘next generation’ sequencing technologies, which still rely on mapping reads to the reference sequence, we would not detect such regions even if we sequenced the whole Y chromosome. However, with third generation sequencing technologies with much longer reads combined with de novo assembly, future work may discover such new sequences, not only on the Y chromosome, but also in the whole genome. Our approach of discovering CNVs by array-CGH limits the precision with which the endpoints can be determined and alternative methods, such as sequenced-based ones (Mills et al. 2011) need to be used for this.

Despite a number of limitations, CNVs affecting protein-coding genes have been effectively discovered in this study, and indeed the well-known common CNVs involving *TSPY* and the gr/gr and b1/b3 regions expected to be present were all detected. Because of the limited phenotypes associated with complete loss or duplication of the entire Y chromosome, the chance of actionable incidental findings from Y studies is low, but some of the variants discovered have potential implications for spermatogenesis.

## Conclusions

We have analysed the distribution of Y-chromosomal CNVs in apparently healthy UK males. Although there are limitations to our dataset, including low sensitivity to small events and a resulting bias towards detecting large CNVs, we show that Y-CNVs can readily be detected. We confirm the abundance of this form of variation on the Y chromosome, where over 6 Mb of sequence is copy number variable and affects over one-third of the male-specific Y proteins. Novel CNVs, both common and rare, continue to be discovered and some of these may have implications for phenotypes, especially spermatogenesis.

## Electronic supplementary material

Supplementary material 1 (PDF 2187 kb)

Supplementary material 2 (PDF 133 kb)

Supplementary material 3 (XLSX 43 kb)

Supplementary material 4 (XLSX 169 kb)
