# Supplementary material for: Copy number variation in the human Y chromosome in the UK population
Source: Hum Genet. 2015 May 10;134(7):789–800. doi: 10.1007/s00439-015-1562-5 (PMC4460274; doi:10.1007/s00439-015-1562-5)
Supplement: Supplementary file 1 — Supplementary material 1 (PDF 2187 kb) [file 439_2015_1562_MOESM1_ESM.pdf]

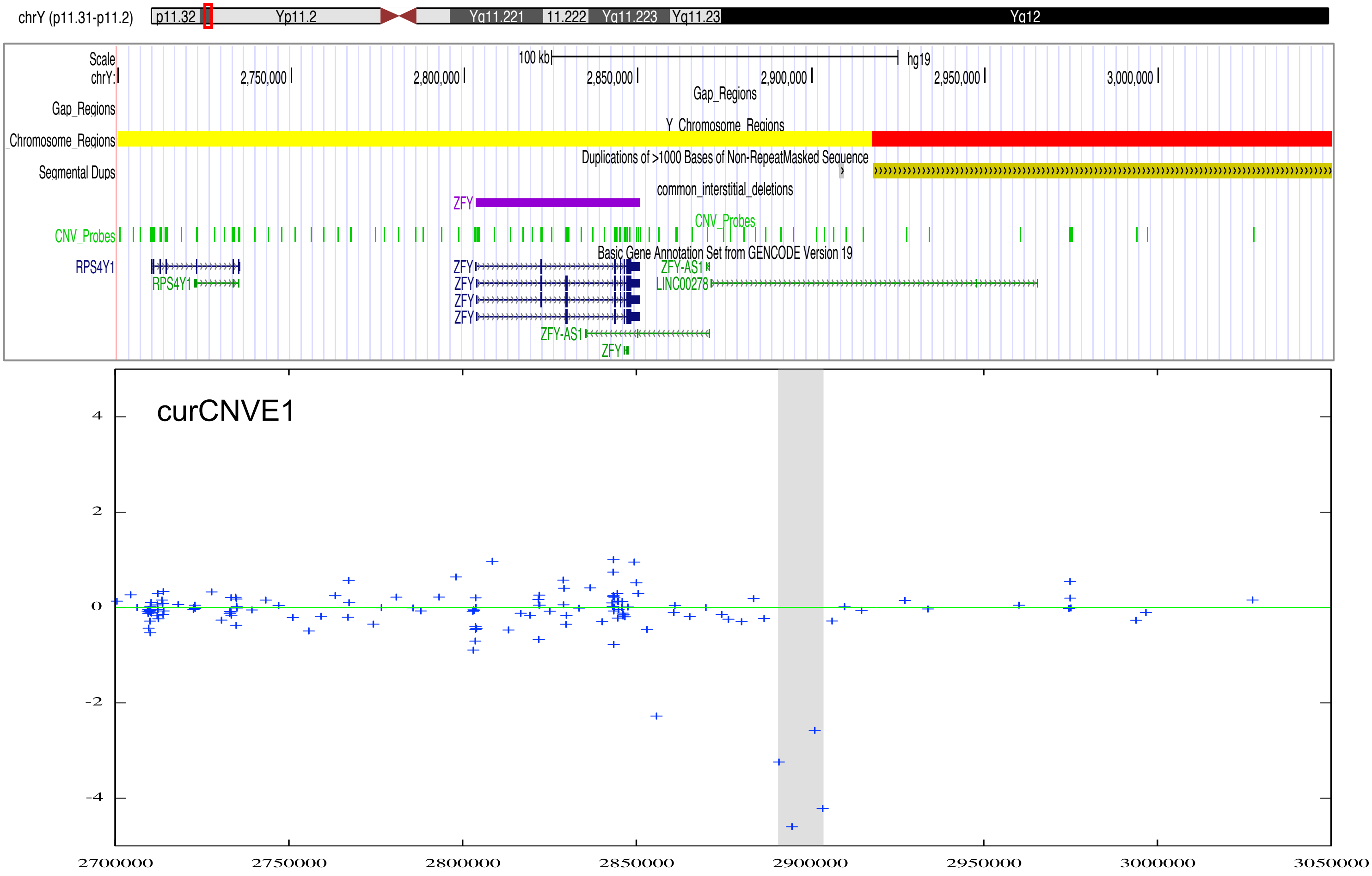

chrY (p11.31-p11.2)
p11.32
Yp11.2
Yq11.221
11.222
Yq11.223
Yq11.23
Yq12
Scale
100 kb
hg19
chrY:
2,750,000
2,800,000
2,850,000
2,900,000
2,950,000
3,000,000
Gap_Regions
Y_Chromosome_Regions
_Chromosome_Regions
Duplications of >1000 Bases of Non-RepeatMasked Sequence
Segmental Dups
common_interstitial_deletions
ZFY
CNV_Probes
Basic Gene Annotation Set from GENCODE Version 19
RPS4Y1
ZFY
ZFY-AS1
LINC00278
curCNVE1
4
2
0
-2
-4
2700000
2750000
2800000
2850000
2900000
2950000
3000000
3050000

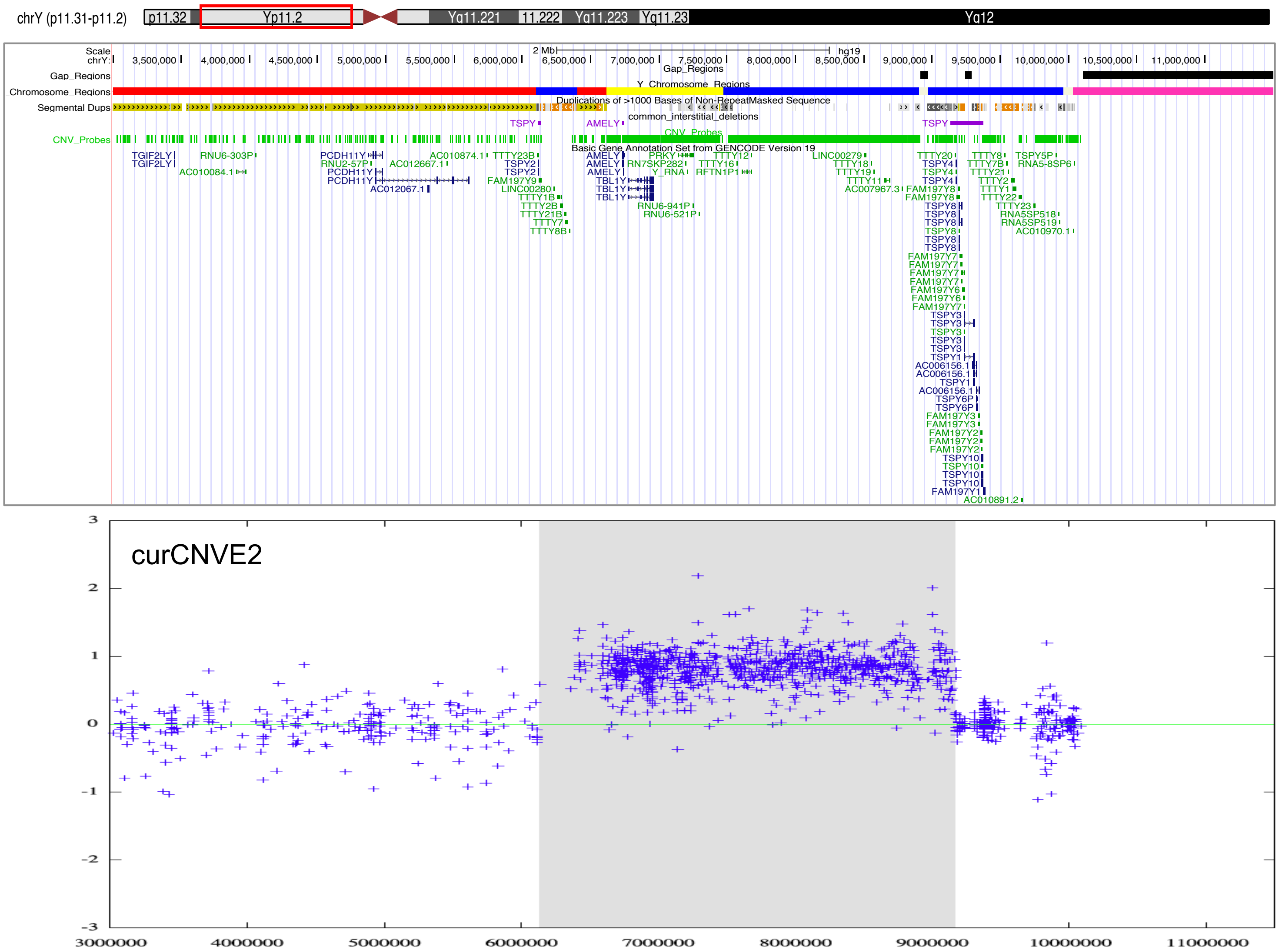
chrY (p11.31-p11.2)
p11.32
Yp11.2
Yq11.221
11.222
Yq11.223
Yq11.23
Yq12
Scale
chrY:
2 Mb
hg19
Gap_Regions
Chromosome_Regions
Y_Chromosome_Regions
Segmental Dups
Duplications of >1000 Bases of Non-RepeatMasked Sequence
common_interstitial_deletions
TSPY
AMELY
CNV_Probes
Basic Gene Annotation Set from GENCODE Version 19
curCNVE2
3000000
4000000
5000000
6000000
7000000
8000000
9000000
10000000
11000000

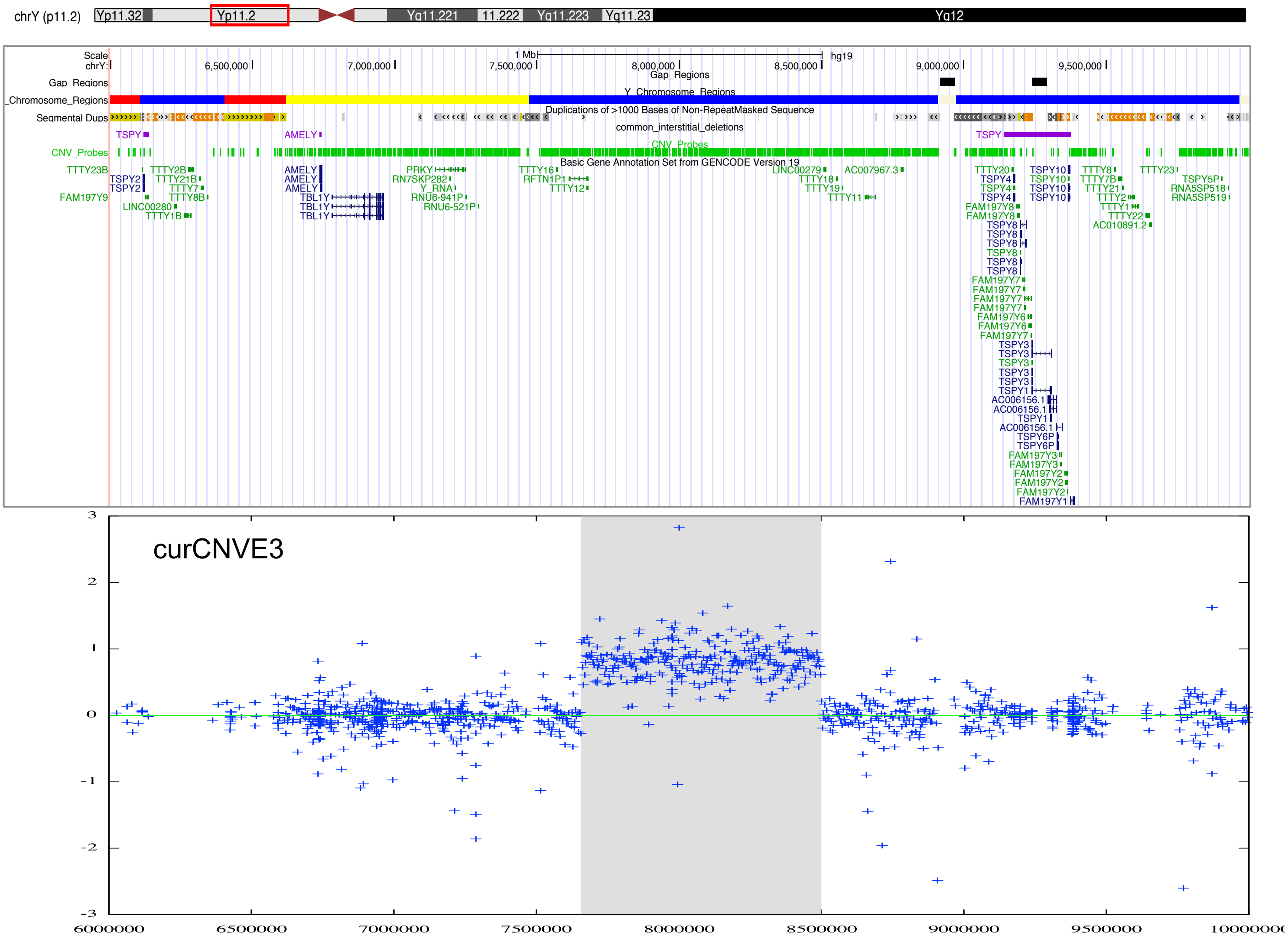
chrY (p11.2)
Yp11.32
Yp11.2
Yq11.221
11.222
Yq11.223
Yq11.23
Yq12
Scale
chrY:
1 Mb
hg19
6,500,000
7,000,000
7,500,000
8,000,000
8,500,000
9,000,000
9,500,000
Gap_Regions
Y_Chromosome_Regions
Segmental Dups
Duplications of >1000 Bases of Non-RepeatMasked Sequence
common_interstitial_deletions
CNV_Probes
Basic Gene Annotation Set from GENCODE Version 19
TSPY
AMELY
curCNVE3
3
2
1
0
-1
-2
-3
6000000
6500000
7000000
7500000
8000000
8500000
9000000
9500000

TSPY Region

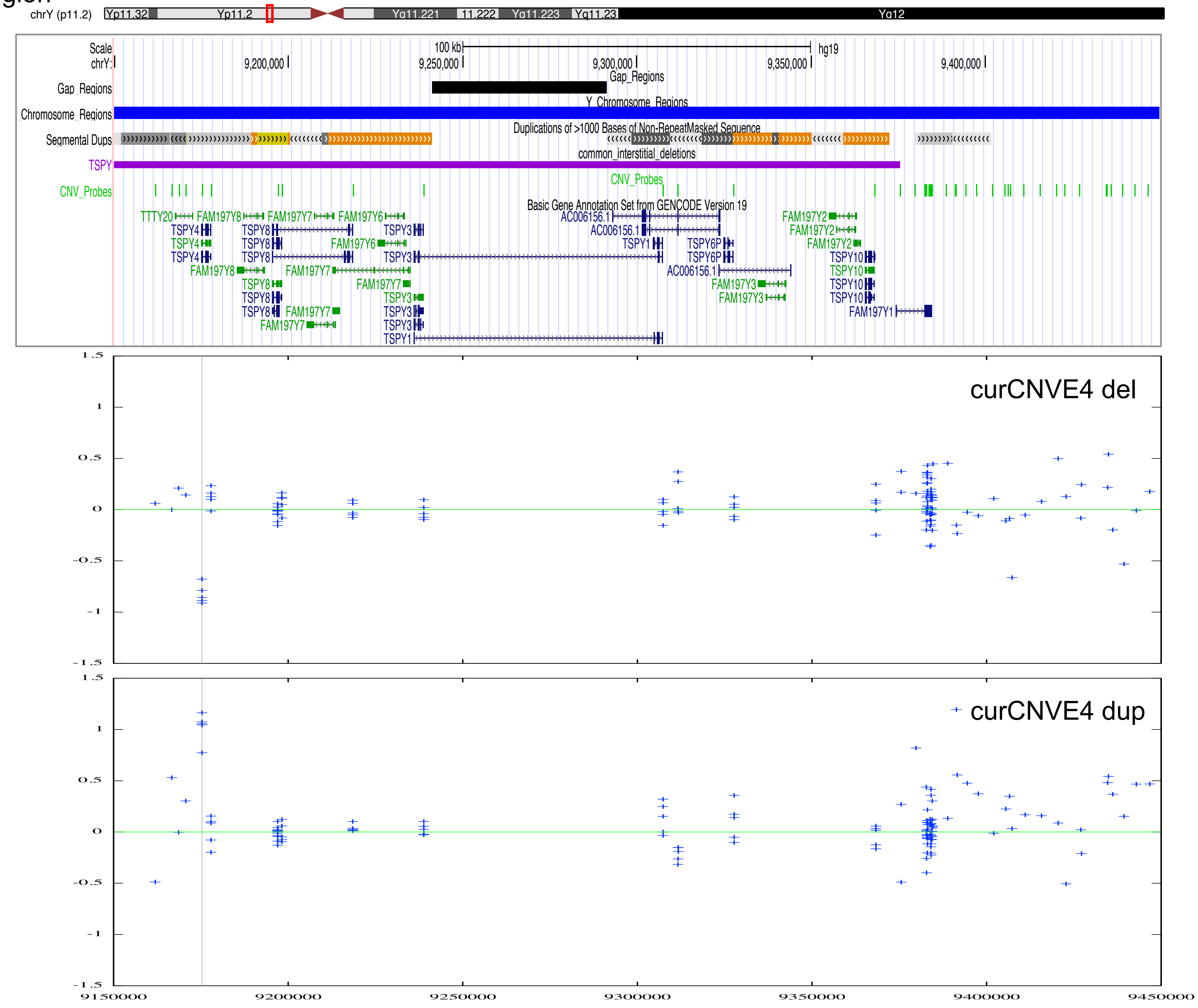

chrY (p11.2)
Yp11.32
Yp11.2
Yq11.221
11.222
Yq11.223
Yq11.23
Yq12
Scale
chrY:
100 kb
hg19
9,200,000
9,250,000
9,300,000
9,350,000
9,400,000
Gap_Regions
Chromosome_Regions
Y_Chromosome_Regions
Segmental Dups
Duplications of >1000 Bases of Non-RepeatMasked Sequence
TSPY
common_interstitial_deletions
CNV_Probes
Basic Gene Annotation Set from GENCODE Version 19
TTTY20
FAM197Y8
FAM197Y7
FAM197Y6
TSPY4
TSPY8
TSPY3
FAM197Y2
TSPY1
TSPY6P
TSPY10
AC006156.1
FAM197Y3
FAM197Y1
curCNVE4 del
curCNVE4 dup
1.5
1
0.5
0
-0.5
-1
-1.5
9150000
9200000
9250000
9300000
9350000
9400000
9450000

TSPY Region

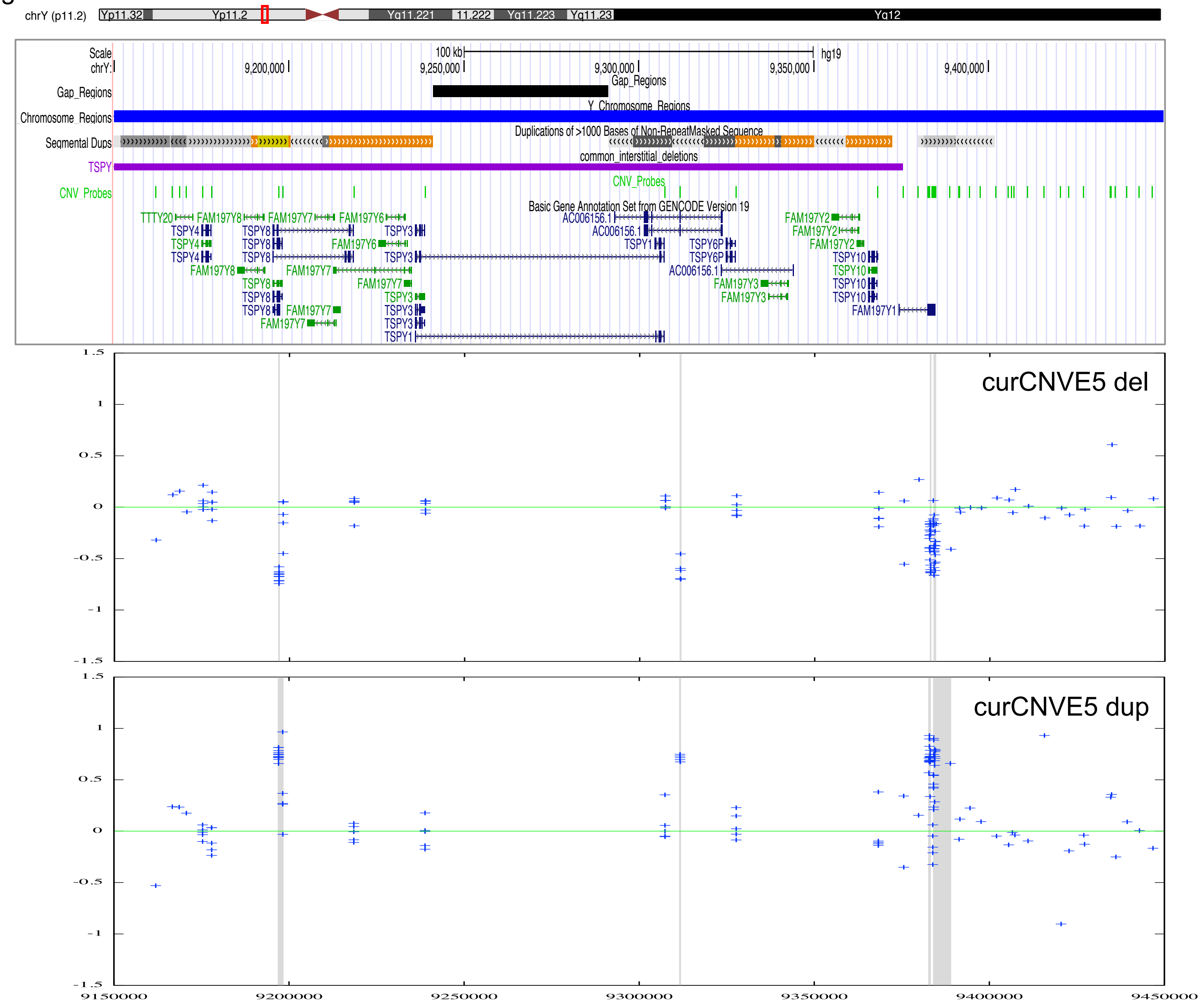
chrY (p11.2)
Yp11.32
Yp11.2
Yq11.221
11.222
Yq11.223
Yq11.23
Yq12
Scale
chrY:
100 kb
hg19
9,200,000
9,250,000
9,300,000
9,350,000
9,400,000
Gap_Regions
Chromosome_Regions
Y_Chromosome_Regions
Segmental Dups
Duplications of >1000 Bases of Non-RepeatMasked Sequence
TSPY
common_interstitial_deletions
CNV_Probes
Basic Gene Annotation Set from GENCODE Version 19
curCNVE5 del
curCNVE5 dup
9150000
9200000
9250000
9300000
9350000
9400000
9450000

TSPY Region

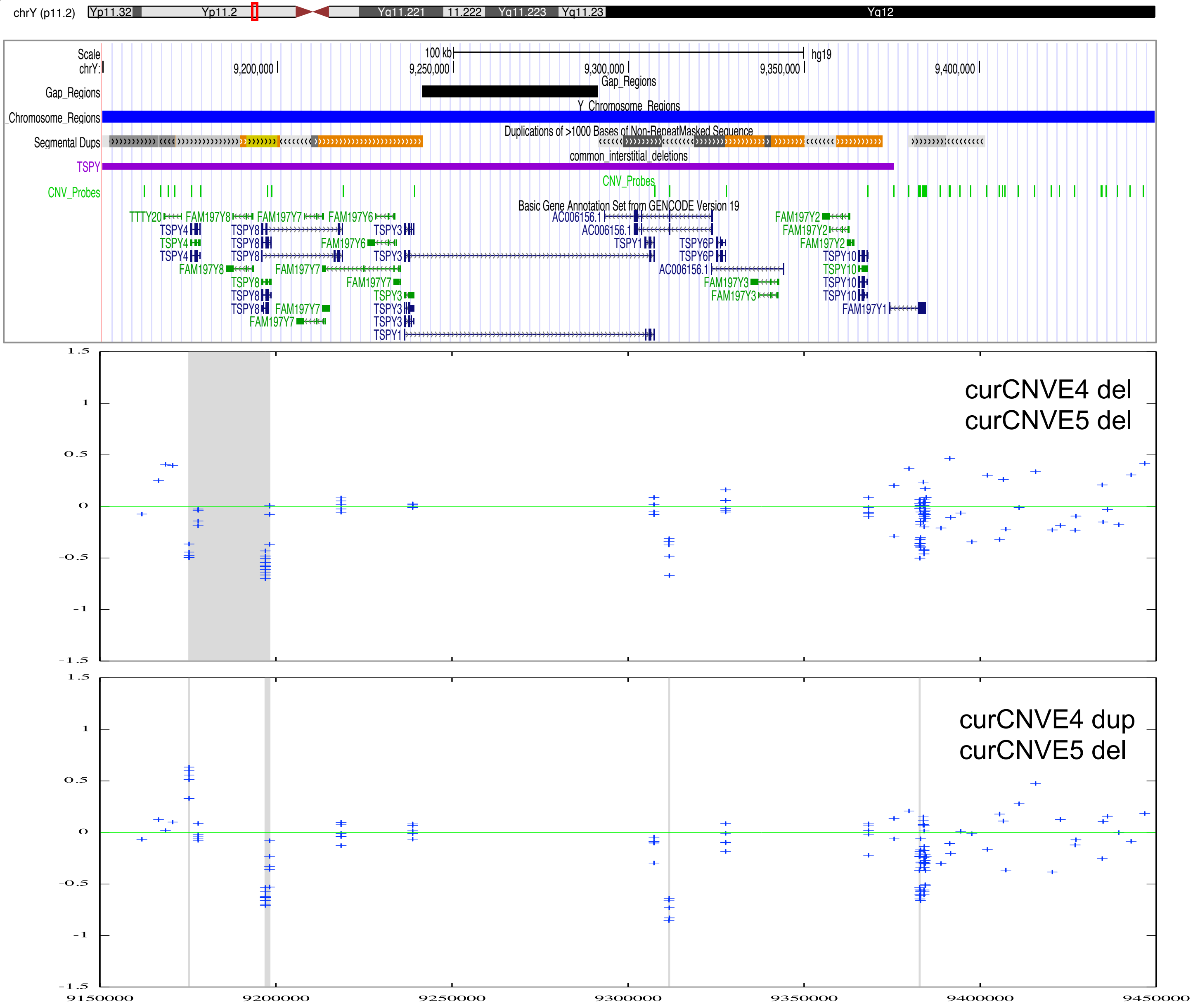

chrY (p11.2)
Yp11.32
Yp11.2
Yq11.221
11.222
Yq11.223
Yq11.23
Yq12
Scale
chrY:
100 kb
hg19
9,200,000
9,250,000
9,300,000
9,350,000
9,400,000
Gap_Regions
Y_Chromosome_Regions
Segmental Dups
Duplications of >1000 Bases of Non-RepeatMasked Sequence
common_interstitial_deletions
TSPY
CNV_Probes
Basic Gene Annotation Set from GENCODE Version 19
TTTY20
TSPY4
FAM197Y8
TSPY8
FAM197Y7
FAM197Y6
TSPY3
TSPY1
AC006156.1
TSPY6P
FAM197Y3
FAM197Y2
TSPY10
FAM197Y1
curCNVE4 del
curCNVE5 del
curCNVE4 dup
curCNVE5 del
1.5
1
0.5
0
-0.5
-1
-1.5
9150000
9200000
9250000
9300000
9350000
9400000
9450000

TSPY Region

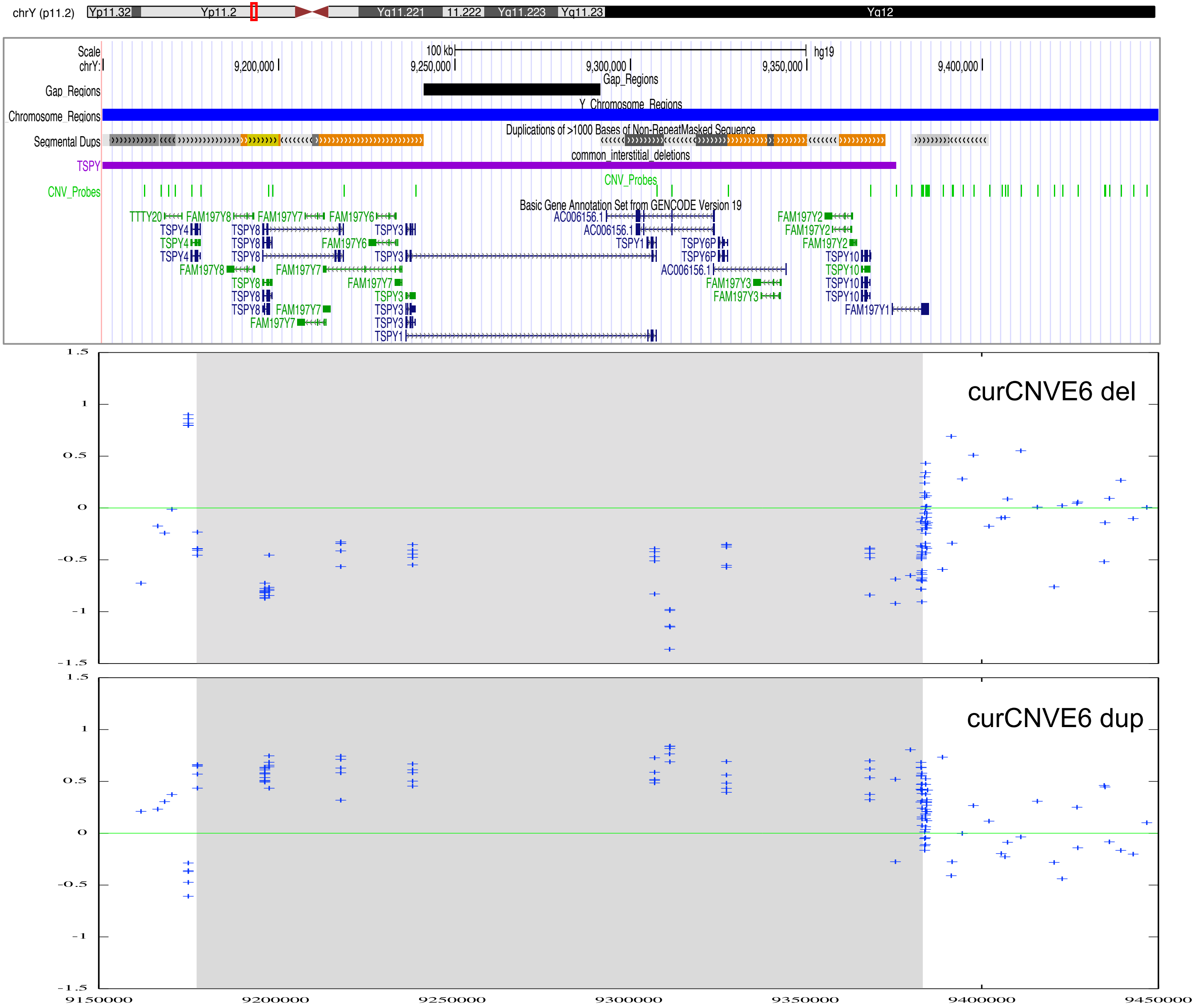

chrY (p11.2)
Yp11.32
Yp11.2
Yq11.221
11.222
Yq11.223
Yq11.23
Yq12
Scale
chrY:
100 kb
hg19
9,200,000
9,250,000
9,300,000
9,350,000
9,400,000
Gap_Regions
Chromosome_Regions
Y_Chromosome_Regions
Segmental Dups
Duplications of >1000 Bases of Non-RepeatMasked Sequence
TSPY
common_interstitial_deletions
CNV_Probes
Basic Gene Annotation Set from GENCODE Version 19
TTTY20
FAM197Y8
FAM197Y7
FAM197Y6
TSPY4
TSPY8
TSPY3
TSPY1
TSPY6P
TSPY10
AC006156.1
FAM197Y2
FAM197Y3
FAM197Y1
curCNVE6 del
curCNVE6 dup
1.5
1
0.5
0
-0.5
-1
-1.5
9150000
9200000
9250000
9300000
9350000
9400000
9450000

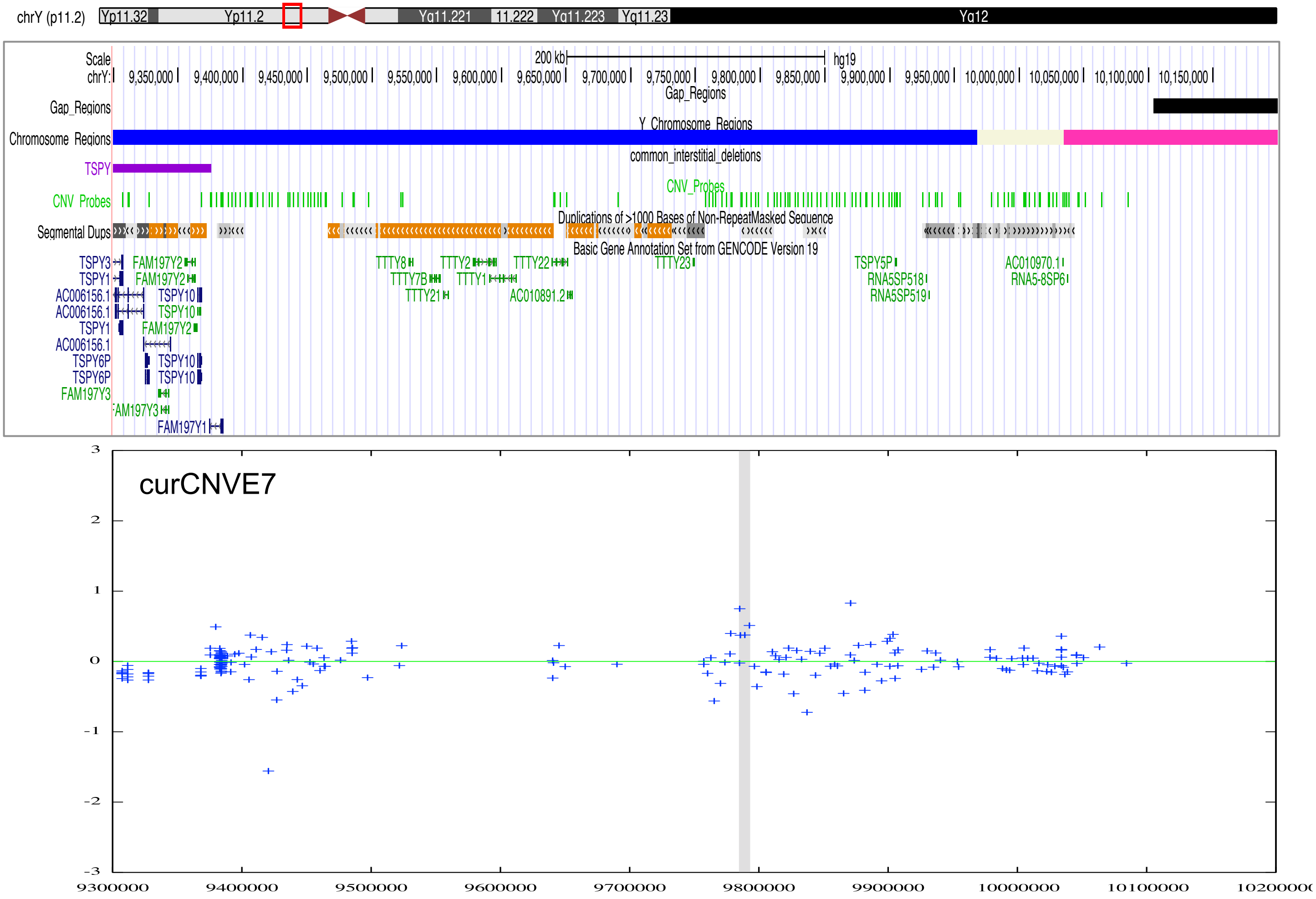

chrY (p11.2)
Yp11.32
Yp11.2
Yq11.221
11.222
Yq11.223
Yq11.23
Yq12
Scale
200 kb
hg19
chrY:
9,350,000
9,400,000
9,450,000
9,500,000
9,550,000
9,600,000
9,650,000
9,700,000
9,750,000
9,800,000
9,850,000
9,900,000
9,950,000
10,000,000
10,050,000
10,100,000
10,150,000
Gap_Regions
Chromosome_Regions
Y_Chromosome_Regions
common_interstitial_deletions
TSPY
CNV_Probes
Segmental Dups
Duplications of >1000 Bases of Non-RepeatMasked Sequence
Basic Gene Annotation Set from GENCODE Version 19
TSPY3
TSPY1
AC006156.1
TSPY6P
FAM197Y3
FAM197Y2
FAM197Y1
TSPY10
TTTY8
TTTY7B
TTTY21
TTTY2
TTTY1
TTTY22
AC010891.2
TTTY23
TSPY5P
RNA5SP518
RNA5SP519
AC010970.1
RNA5-8SP6
curCNVE7
3
2
1
0
-1
-2
-3
9300000
9400000
9500000
9600000
9700000
9800000
9900000
10000000
10100000

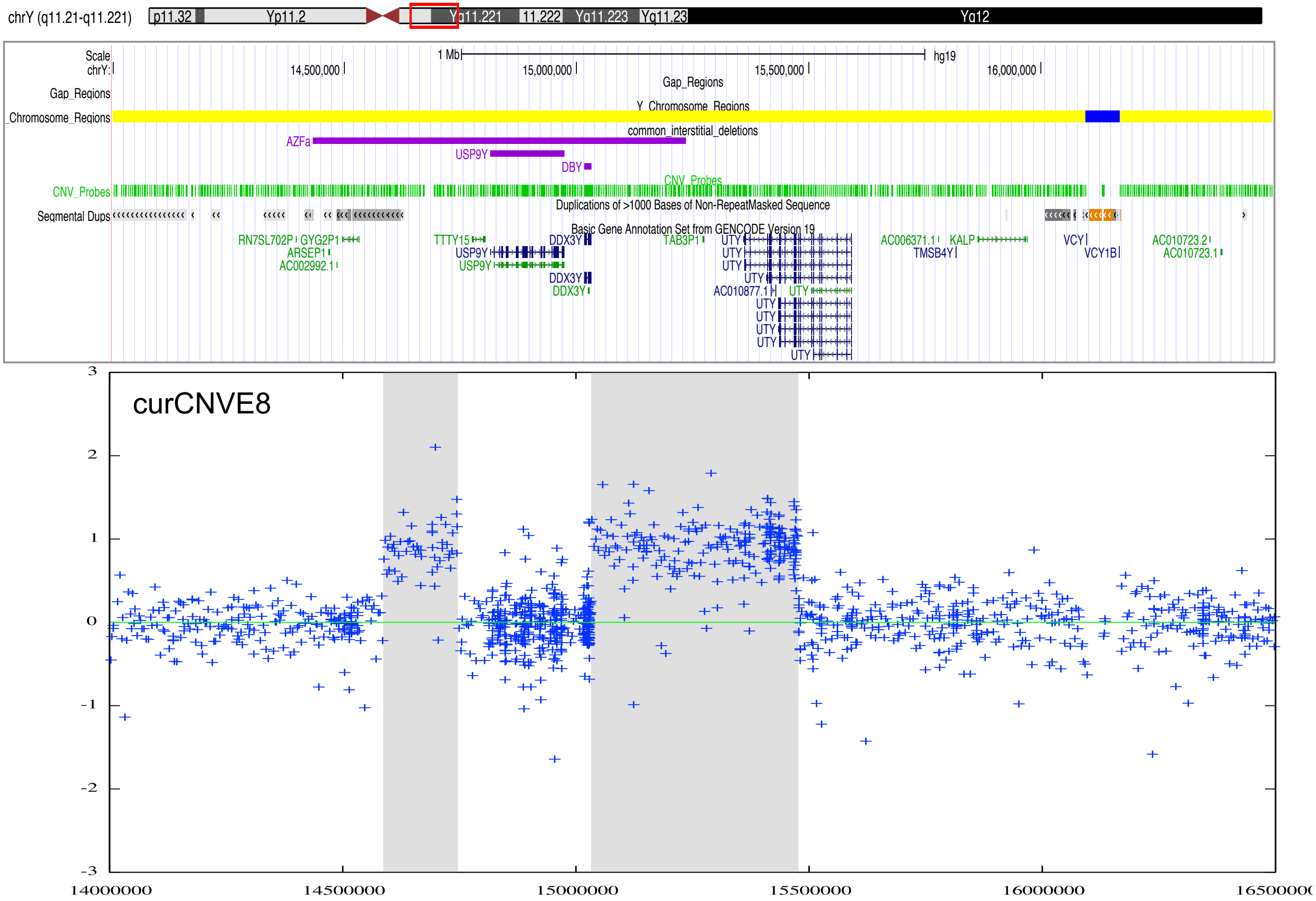

chrY (q11.21-q11.221)
p11.32
Yp11.2
Yq11.221
11.222
Yq11.223
Yq11.23
Yq12
Scale
chrY:
1 Mb
hg19
14,500,000
15,000,000
15,500,000
16,000,000
Gap_Regions
Y_Chromosome_Regions
common_interstitial_deletions
AZFa
USP9Y
DBY
CNV_Probes
Segmental Dups
Duplications of >1000 Bases of Non-RepeatMasked Sequence
Basic Gene Annotation Set from GENCODE Version 19
RN7SL702P
GYG2P1
ARSEP1
AC002992.1
TTTY15
USP9Y
DDX3Y
TAB3P1
UTY
AC010877.1
AC006371.1
KALP
TMSB4Y
VCY
VCY1B
AC010723.2
AC010723.1
curCNVE8
3
2
1
0
-1
-2
-3
14000000
14500000
15000000
15500000
16000000

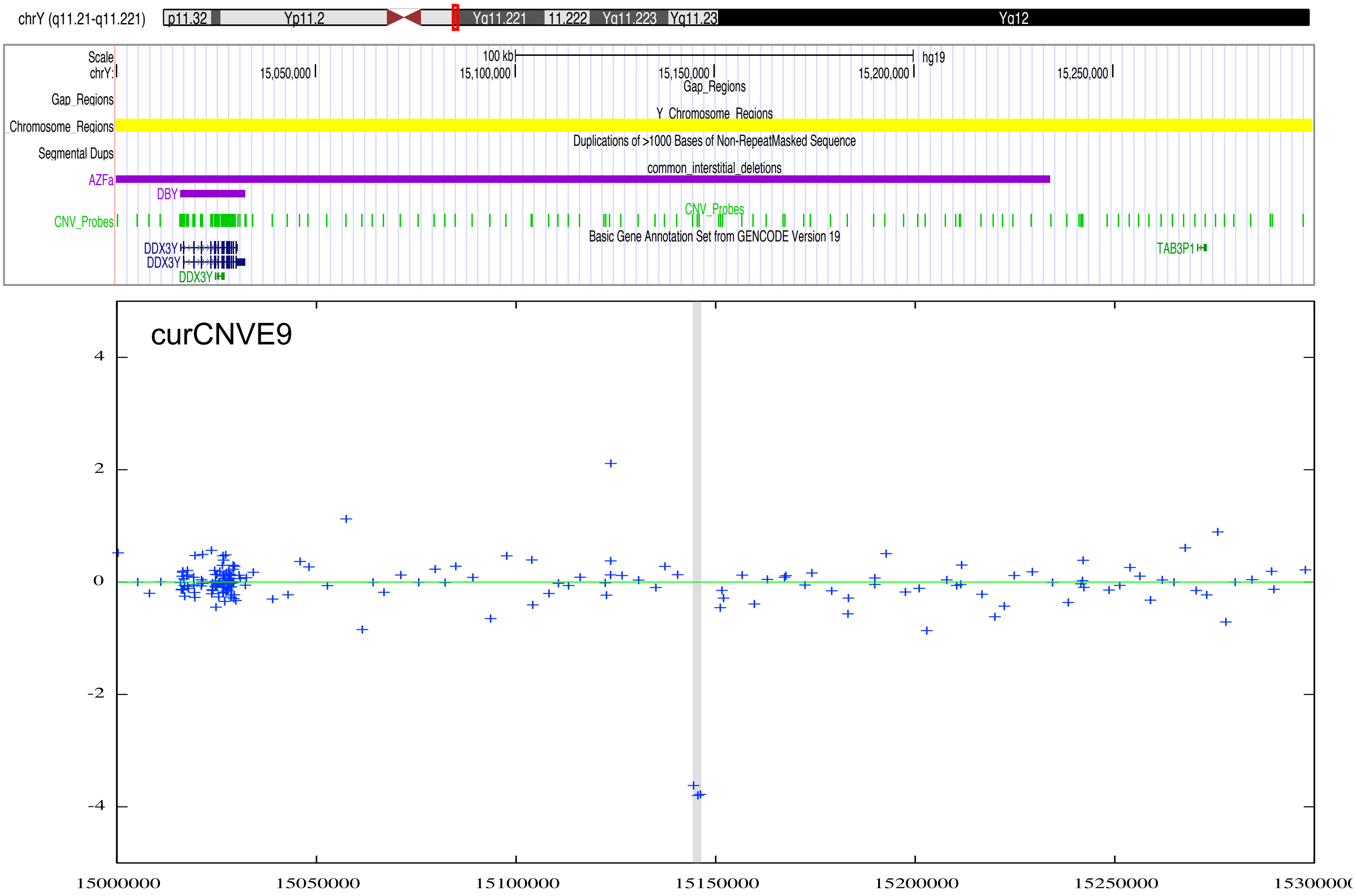

chrY (q11.21-q11.221)
p11.32
Yp11.2
Yq11.221
11.222
Yq11.223
Yq11.23
Yq12
Scale
chrY:
100 kb
hg19
15,050,000
15,100,000
15,150,000
15,200,000
15,250,000
Gap_Regions
Chromosome_Regions
Y_Chromosome_Regions
Duplications of >1000 Bases of Non-RepeatMasked Sequence
Segmental Dups
common_interstitial_deletions
AZFa
DBY
CNV_Probes
Basic Gene Annotation Set from GENCODE Version 19
DDX3Y
TAB3P1
curCNVE9
4
2
0
-2
-4
15000000
15050000
15100000
15150000
15200000
15250000

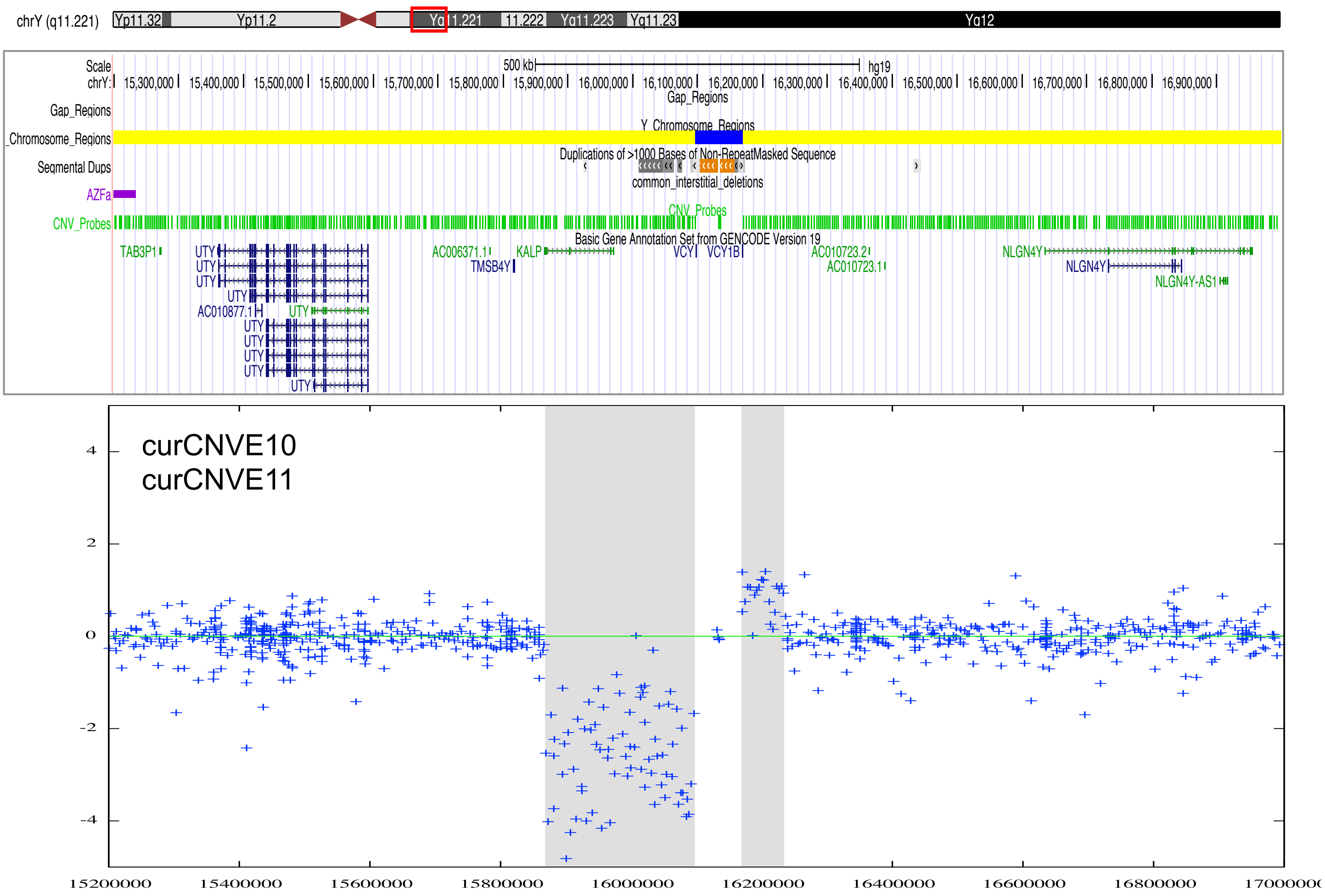
chrY (q11.221)
Yp11.32
Yp11.2
Yq11.221
11.222
Yq11.223
Yq11.23
Yq12
Scale
500 kb
hg19
chrY:
15,300,000
15,400,000
15,500,000
15,600,000
15,700,000
15,800,000
15,900,000
16,000,000
16,100,000
16,200,000
16,300,000
16,400,000
16,500,000
16,600,000
16,700,000
16,800,000
16,900,000
Gap_Regions
Y_Chromosome_Regions
Duplications of >1000 Bases of Non-RepeatMasked Sequence
Segmental Dups
common_interstitial_deletions
AZFa
CNV_Probes
Basic Gene Annotation Set from GENCODE Version 19
TAB3P1
UTY
AC010877.1
AC006371.1
TMSB4Y
KALP
VCY
VCY1B
AC010723.2
AC010723.1
NLGN4Y
NLGN4Y-AS1
curCNVE10
curCNVE11
4
2
0
-2
-4
15200000
15400000
15600000
15800000
16000000
16200000
16400000
16600000
16800000
17000000

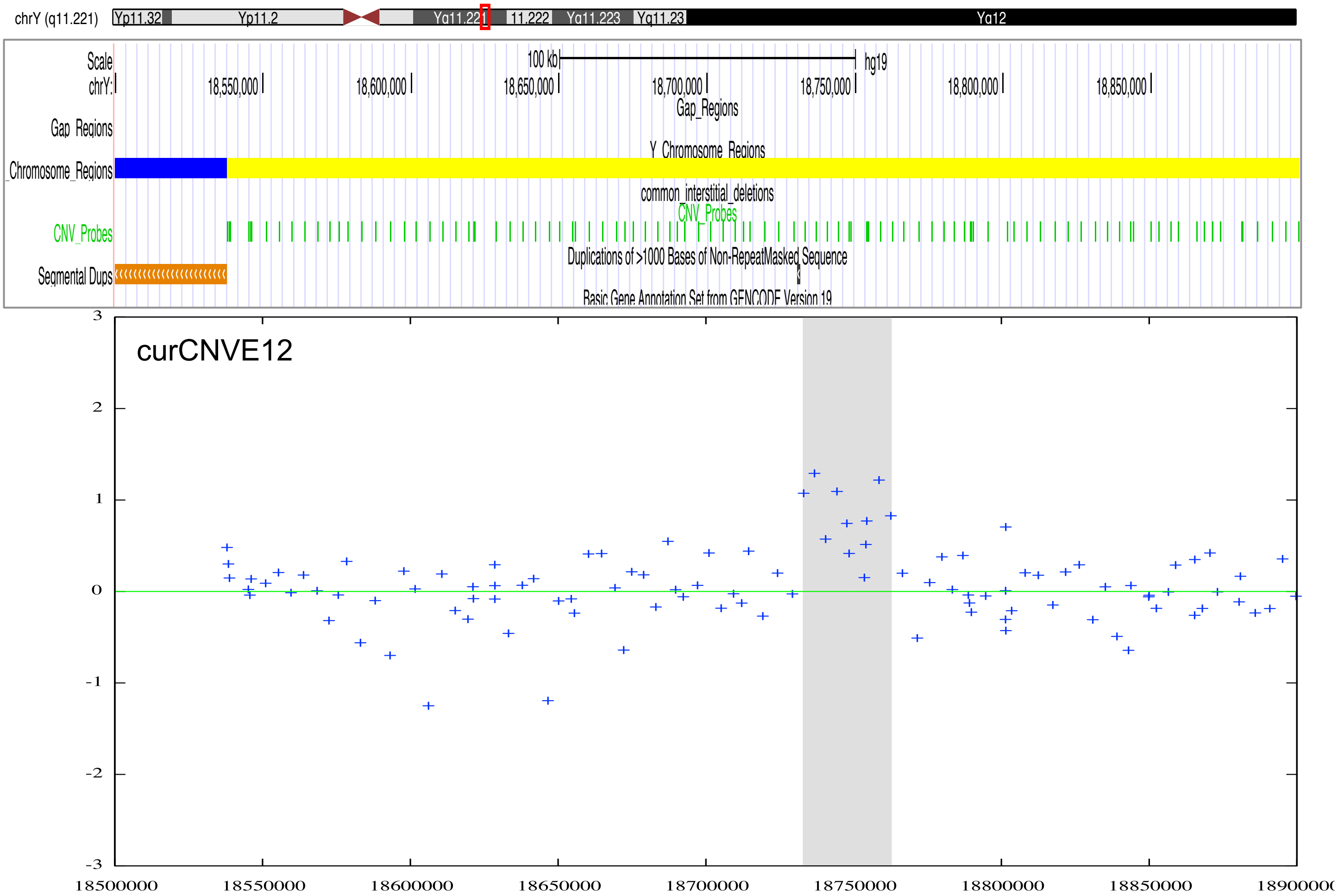
chrY (q11.221)
Yp11.32
Yp11.2
Yq11.221
11.222
Yq11.223
Yq11.23
Yq12
Scale
100 kb
hg19
chrY:
18,550,000
18,600,000
18,650,000
18,700,000
18,750,000
18,800,000
18,850,000
Gap_Regions
Gap_Regions
Y_Chromosome_Regions
Chromosome_Regions
common_interstitial_deletions
CNV_Probes
CNV_Probes
Duplications of >1000 Bases of Non-RepeatMasked Sequence
Segmental Dups
Basic Gene Annotation Set from GENCODE Version 19
curCNVE12
3
2
1
0
-1
-2
-3
18500000
18550000
18600000
18650000
18700000
18750000
18800000
18850000
18900000

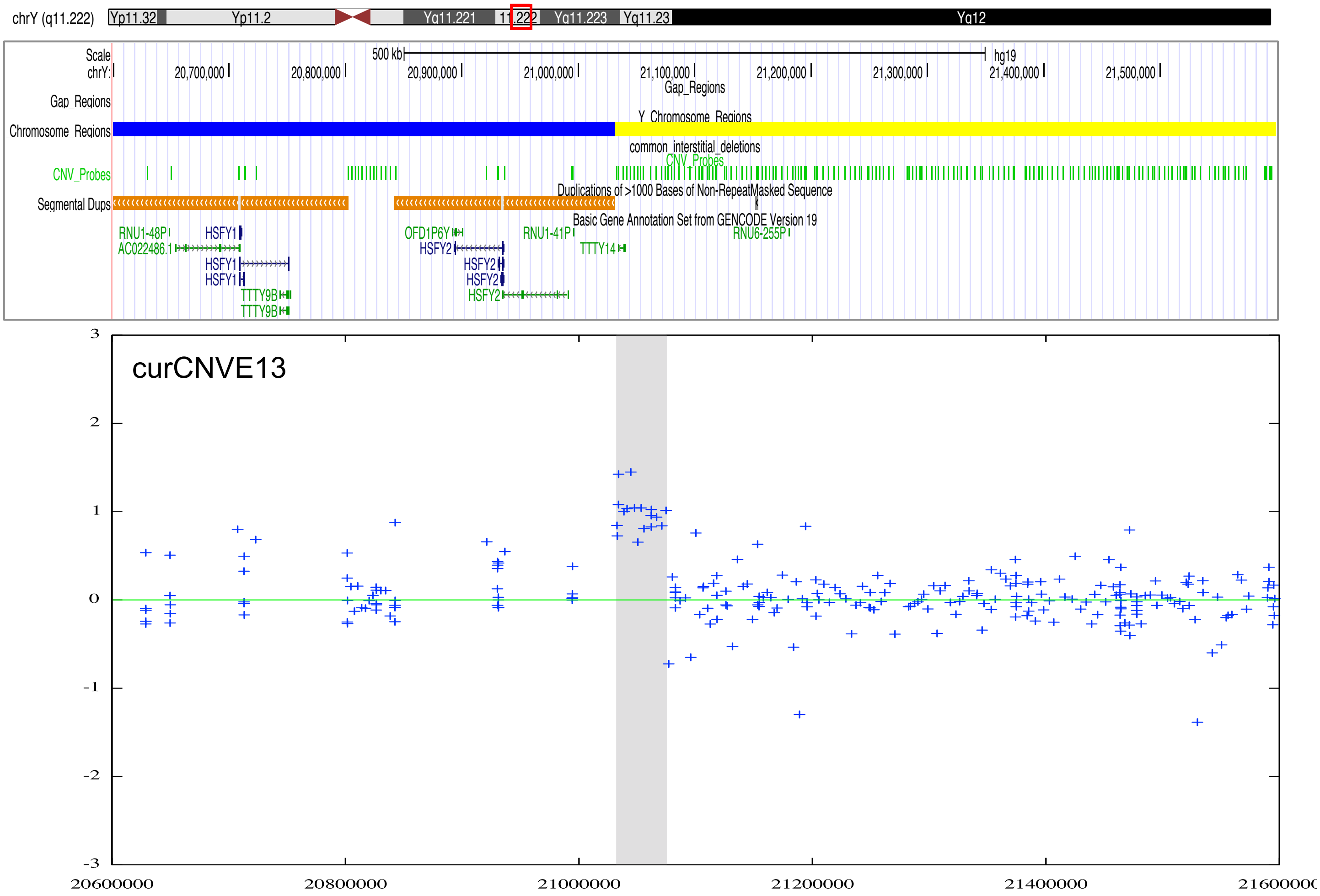
chrY (q11.222)
Yp11.32
Yp11.2
Yq11.221
11.222
Yq11.223
Yq11.23
Yq12
Scale
chrY:
500 kb
hg19
20,700,000
20,800,000
20,900,000
21,000,000
21,100,000
21,200,000
21,300,000
21,400,000
21,500,000
Gap Regions
Gap_Regions
Chromosome Regions
Y Chromosome Regions
common_interstitial_deletions
CNV_Probes
Segmental Dups
Duplications of >1000 Bases of Non-RepeatMasked Sequence
Basic Gene Annotation Set from GENCODE Version 19
RNU1-48P
AC022486.1
HSFY1
TTTY9B
OFD1P6Y
HSFY2
RNU1-41P
TTTY14
RNU6-255P
curCNVE13
3
2
1
0
-1
-2
-3
20600000
20800000
21000000
21200000
21400000
21600000

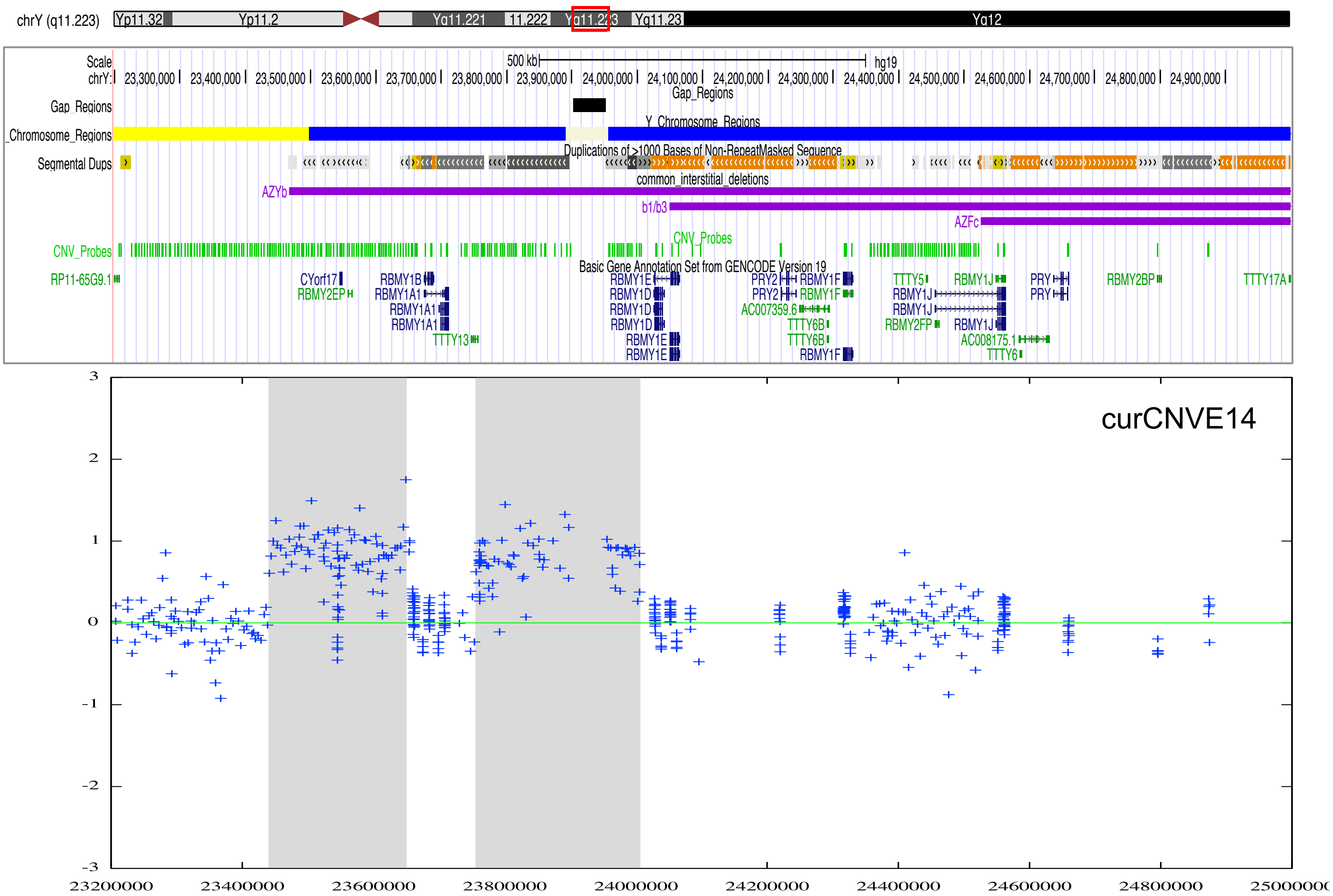
chrY (q11.223)
Yp11.32
Yp11.2
Yq11.221
11.222
Yq11.223
Yq11.23
Yq12
Scale
500 kb
hg19
chrY:
23,300,000
23,400,000
23,500,000
23,600,000
23,700,000
23,800,000
23,900,000
24,000,000
24,100,000
24,200,000
24,300,000
24,400,000
24,500,000
24,600,000
24,700,000
24,800,000
24,900,000
Gap_Regions
Y_Chromosome_Regions
Segmental Dups
Duplications of >1000 Bases of Non-RepeatMasked Sequence
common_interstitial_deletions
AZYb
b1/b3
AZFc
CNV_Probes
Basic Gene Annotation Set from GENCODE Version 19
RP11-65G9.1
CYorf17
RBMY2EP
RBMY1B
RBMY1A1
TTTY13
RBMY1E
RBMY1D
PRY2
RBMY1F
AC007359.6
TTTY6B
TTTY5
RBMY1J
RBMY2FP
AC008175.1
TTTY6
PRY
RBMY2BP
TTTY17A
curCNVE14
3
2
1
0
-1
-2
-3
23200000
23400000
23600000
23800000
24000000
24200000
24400000
24600000
24800000
25000000

RBMY Region

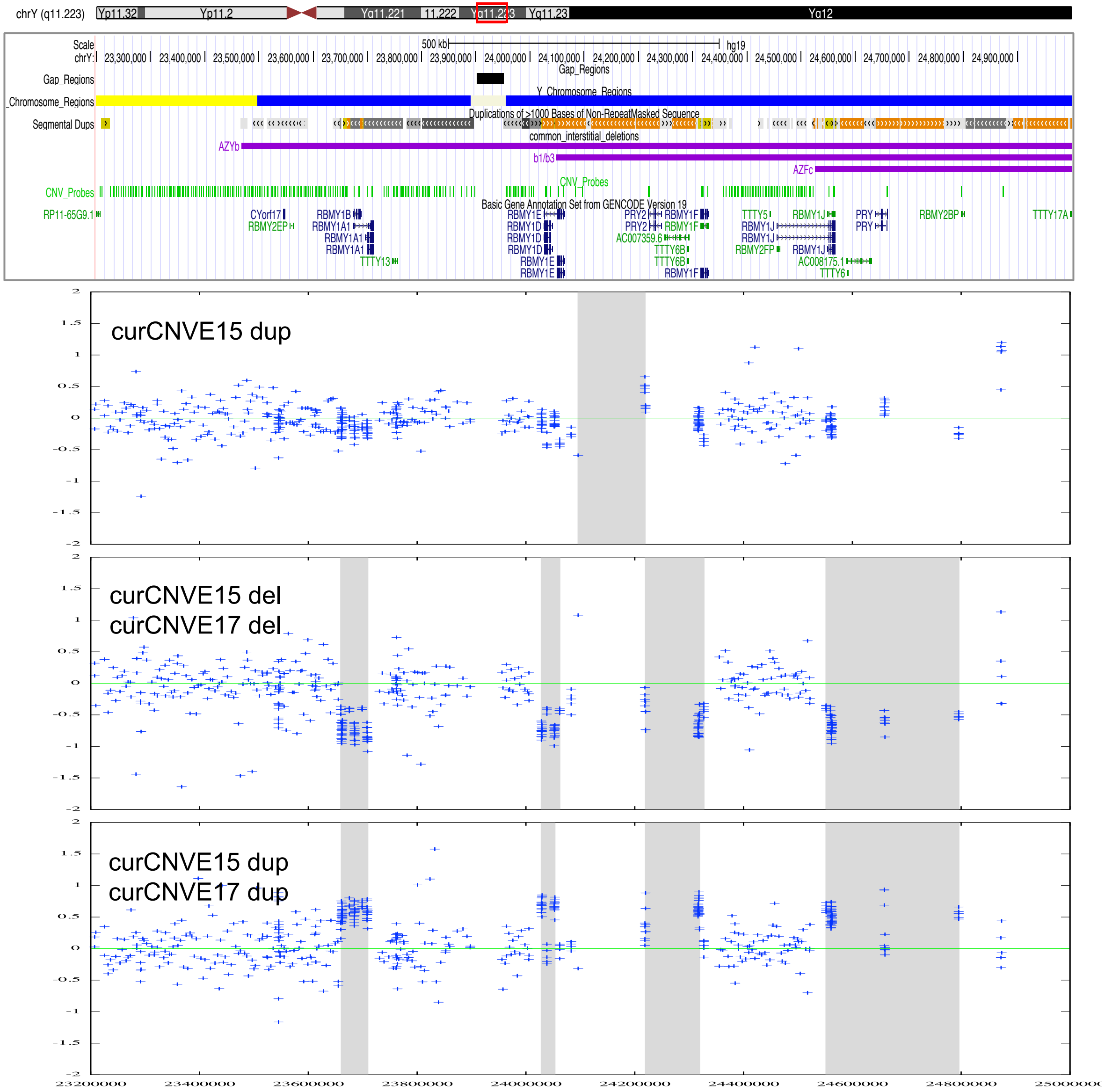

chrY (q11.223)
Yp11.32
Yp11.2
Yq11.221
11.222
Yq11.23
Yq12
Scale
chrY:
500 kb
hg19
23,300,000
23,400,000
23,500,000
23,600,000
23,700,000
23,800,000
23,900,000
24,000,000
24,100,000
24,200,000
24,300,000
24,400,000
24,500,000
24,600,000
24,700,000
24,800,000
24,900,000
Gap_Regions
Y_Chromosome_Regions
Segmental Dups
Duplications of >1000 Bases of Non-RepeatMasked Sequence
common_interstitial_deletions
AZYb
b1/b3
AZFc
CNV_Probes
Basic Gene Annotation Set from GENCODE Version 19
curCNVE15 dup
curCNVE15 del
curCNVE17 del
curCNVE15 dup
curCNVE17 dup
23200000
23400000
23600000
23800000
24000000
24200000
24400000
24600000
24800000

RBMY Region

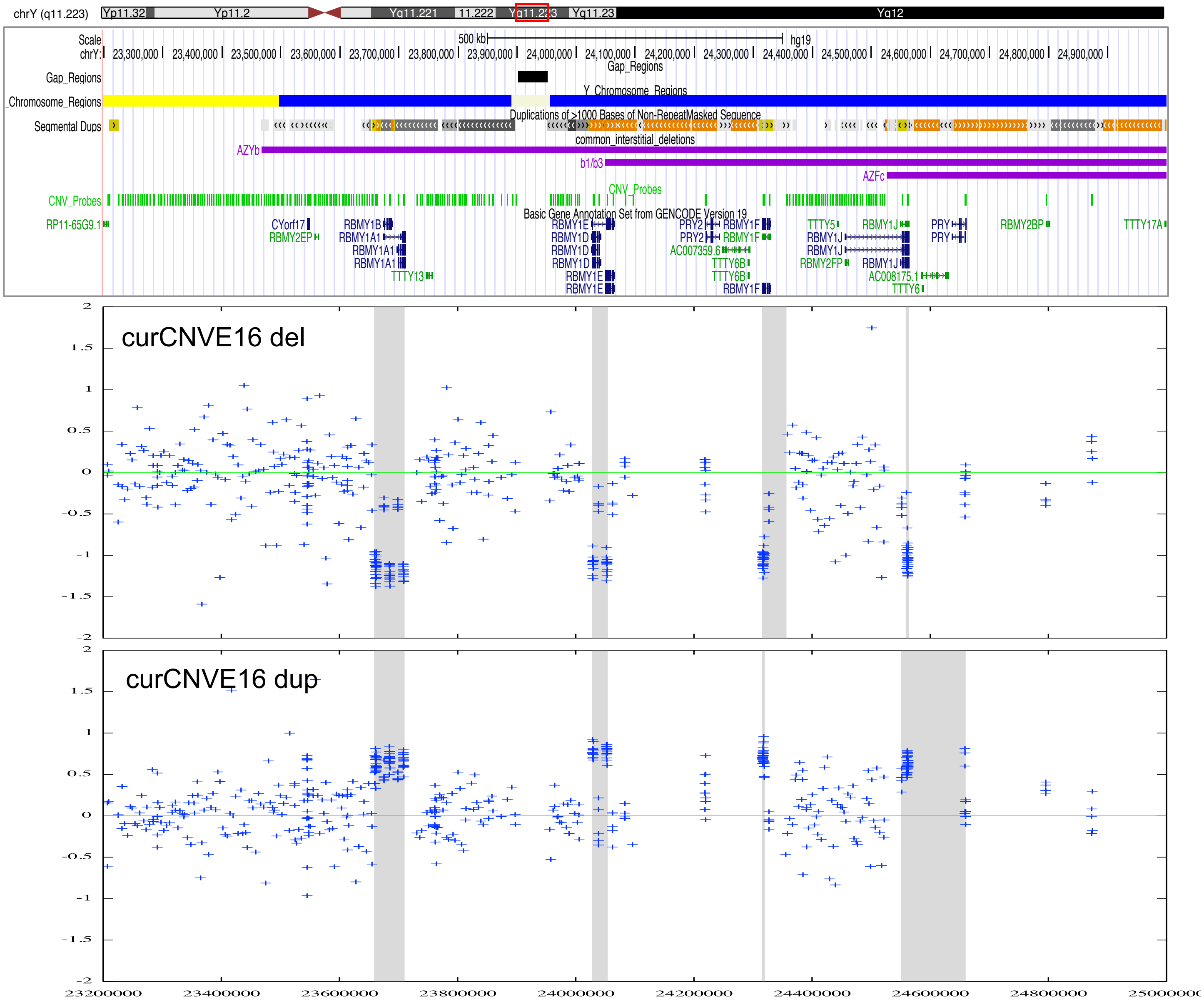

chrY (q11.223)
Yp11.32
Yp11.2
Yq11.221
11.222
Yq11.223
Yq11.23
Yq12
Scale
500 kb
hg19
chrY:
Gap_Regions
Y_Chromosome_Regions
Segmental Dups
Duplications of >1000 Bases of Non-RepeatMasked Sequence
common_interstitial_deletions
AZYb
b1/b3
AZFc
CNV_Probes
Basic Gene Annotation Set from GENCODE Version 19
curCNVE16 del
curCNVE16 dup

RBMY Region

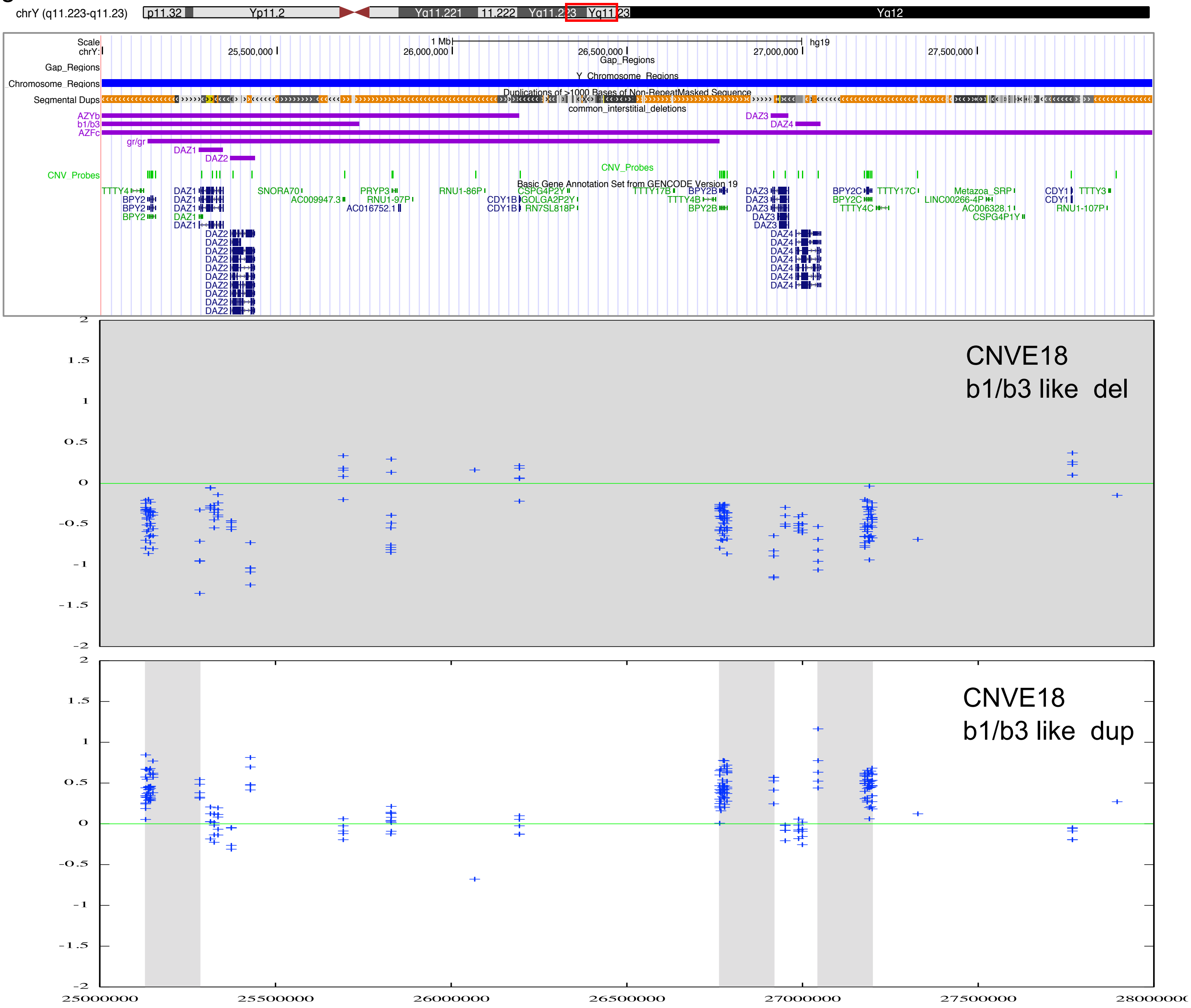
chrY (q11.223-q11.23)
p11.32
Yp11.2
Yq11.221
11.222
Yq11.223
Yq12
Scale
chrY:
1 Mb
hg19
25,500,000
26,000,000
26,500,000
27,000,000
27,500,000
Gap_Regions
Chromosome_Regions
Y_Chromosome_Regions
Segmental Dups
Duplications of >1000 Bases of Non-RepeatMasked Sequence
common_interstitial_deletions
AZYb
b1/b3
AZFc
gr/gr
DAZ1
DAZ2
DAZ3
DAZ4
CNV_Probes
Basic Gene Annotation Set from GENCODE Version 19
TTTY4
BPY2
SNORA70
AC009947.3
PRYP3
RNU1-97P
AC016752.1
RNU1-86P
CDY1B
CSPG4P2Y
GOLGA2P2Y
RN7SL818P
TTTY17B
TTTY4B
BPY2B
BPY2C
TTTY17C
TTTY4C
Metazoa_SRP
LINC00266-4P
AC006328.1
CSPG4P1Y
CDY1
TTTY3
RNU1-107P
CNVE18
b1/b3 like del
CNVE18
b1/b3 like dup
2
1.5
1
0.5
0
-0.5
-1
-1.5
-2
25000000
25500000
26000000
26500000
27000000
27500000
28000000

DAZ Region

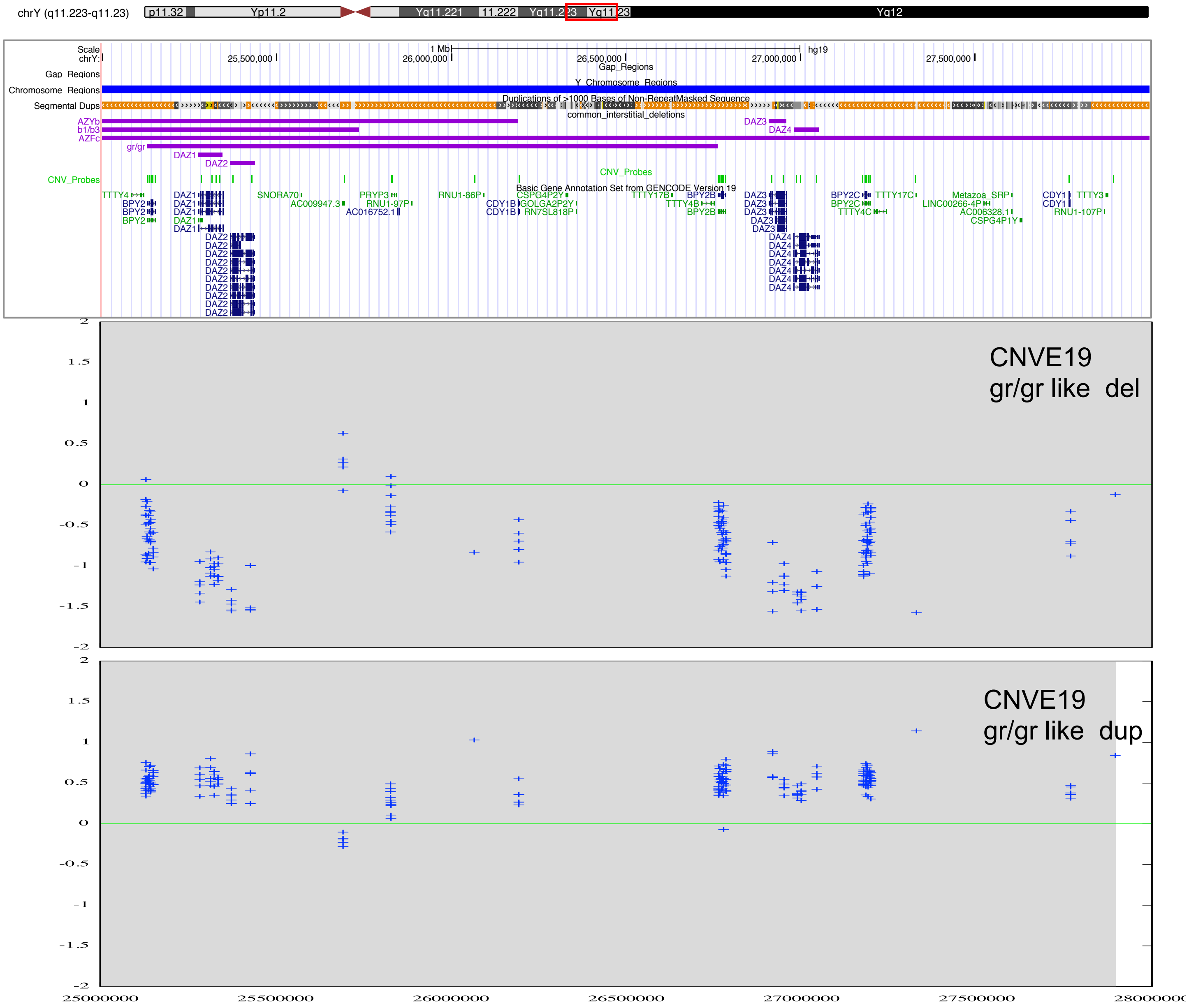

DAZ Region

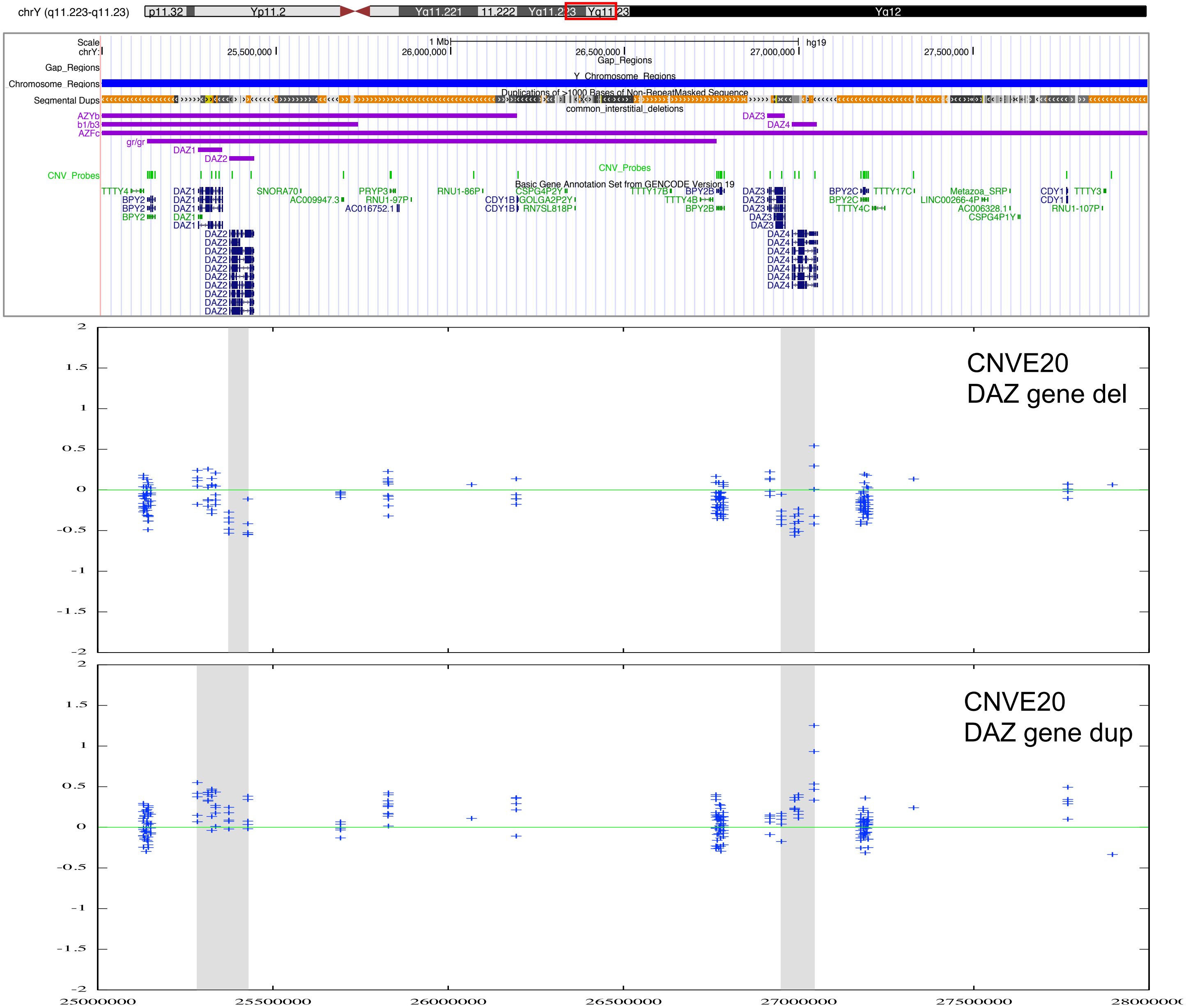
chrY (q11.223-q11.23)
Scale
chrY:
25,500,000
26,000,000
26,500,000
27,000,000
27,500,000
1 Mb
hg19
Gap_Regions
Chromosome_Regions
Y_Chromosome_Regions
Segmental Dups
Duplications of >1000 Bases of Non-RepeatMasked Sequence
common_interstitial_deletions
AZYb
b1/b3
AZFc
gr/gr
DAZ1
DAZ2
DAZ3
DAZ4
CNV_Probes
Basic Gene Annotation Set from GENCODE Version 19
CNVE20
DAZ gene del
CNVE20
DAZ gene dup
25000000
25500000
26000000
26500000
27000000
27500000
28000000

DAZ Region

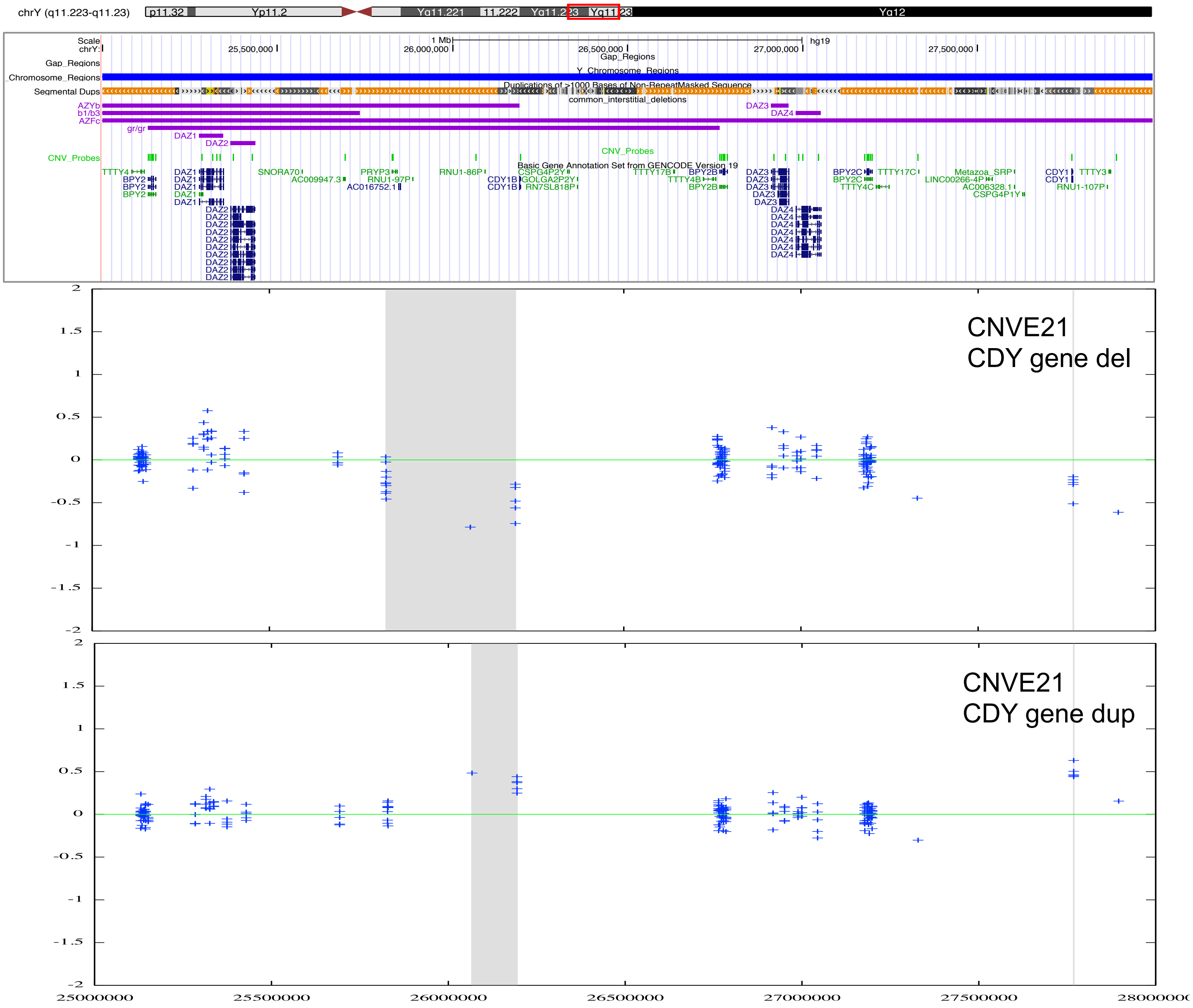

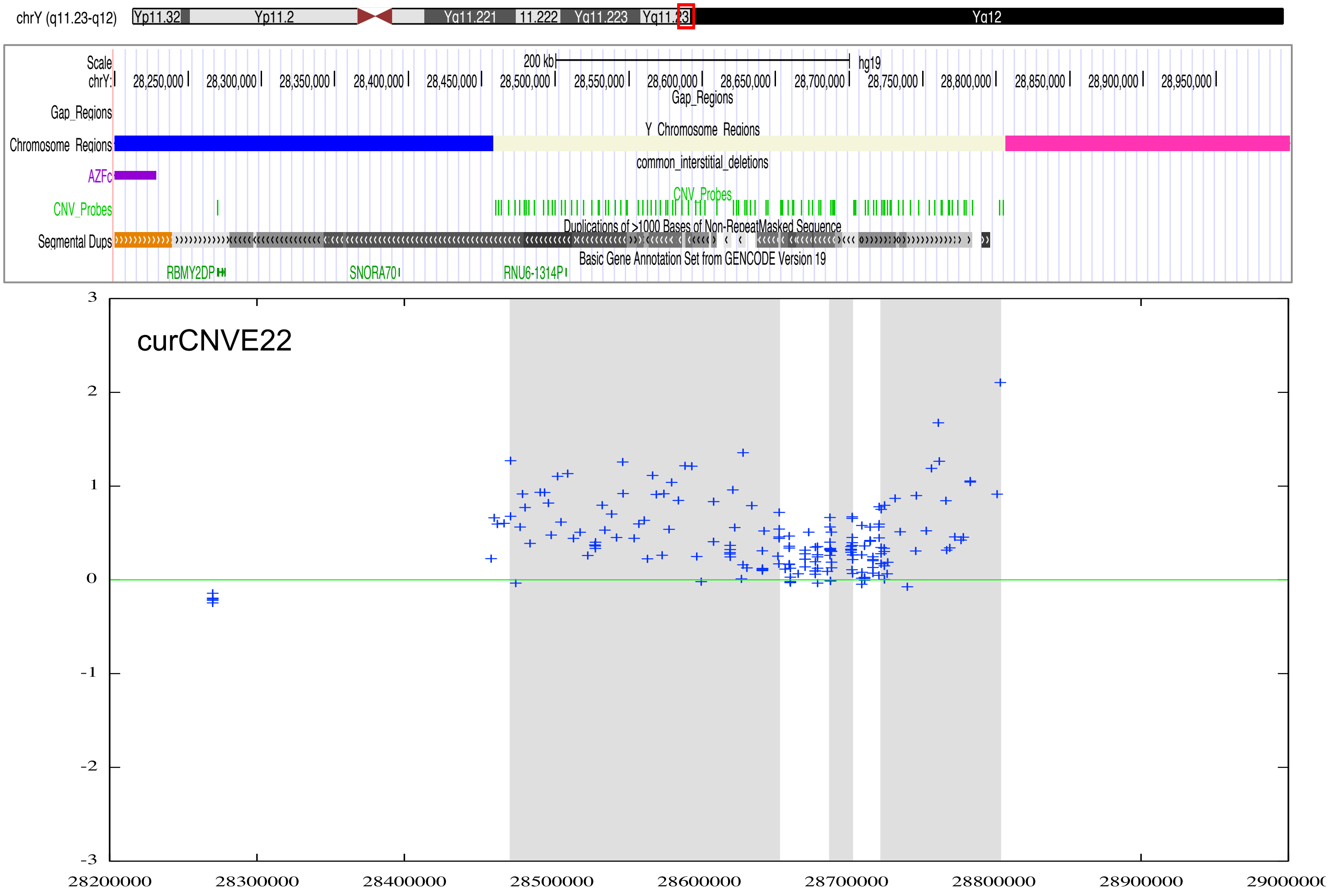
chrY (q11.23-q12)
Yp11.32
Yp11.2
Yq11.221
11.222
Yq11.223
Yq11.23
Yq12
Scale
chrY:
200 kb
hg19
28,250,000
28,300,000
28,350,000
28,400,000
28,450,000
28,500,000
28,550,000
28,600,000
28,650,000
28,700,000
28,750,000
28,800,000
28,850,000
28,900,000
28,950,000
Gap_Regions
Chromosome_Regions
Y_Chromosome_Regions
AZFc
common_interstitial_deletions
CNV_Probes
Segmental Dups
Duplications of >1000 Bases of Non-RepeatMasked Sequence
Basic Gene Annotation Set from GENCODE Version 19
RBMY2DP
SNORA70
RNU6-1314P
curCNVE22
3
2
1
0
-1
-2
-3
28200000
28300000
28400000
28500000
28600000
28700000
28800000
28900000
29000000
