# Supplementary figures and images for: Copy number variation in the human Y chromosome in the UK population
Source: Hum Genet. 2015 May 10;134(7):789–800. doi: 10.1007/s00439-015-1562-5 (PMC4460274; doi:10.1007/s00439-015-1562-5)

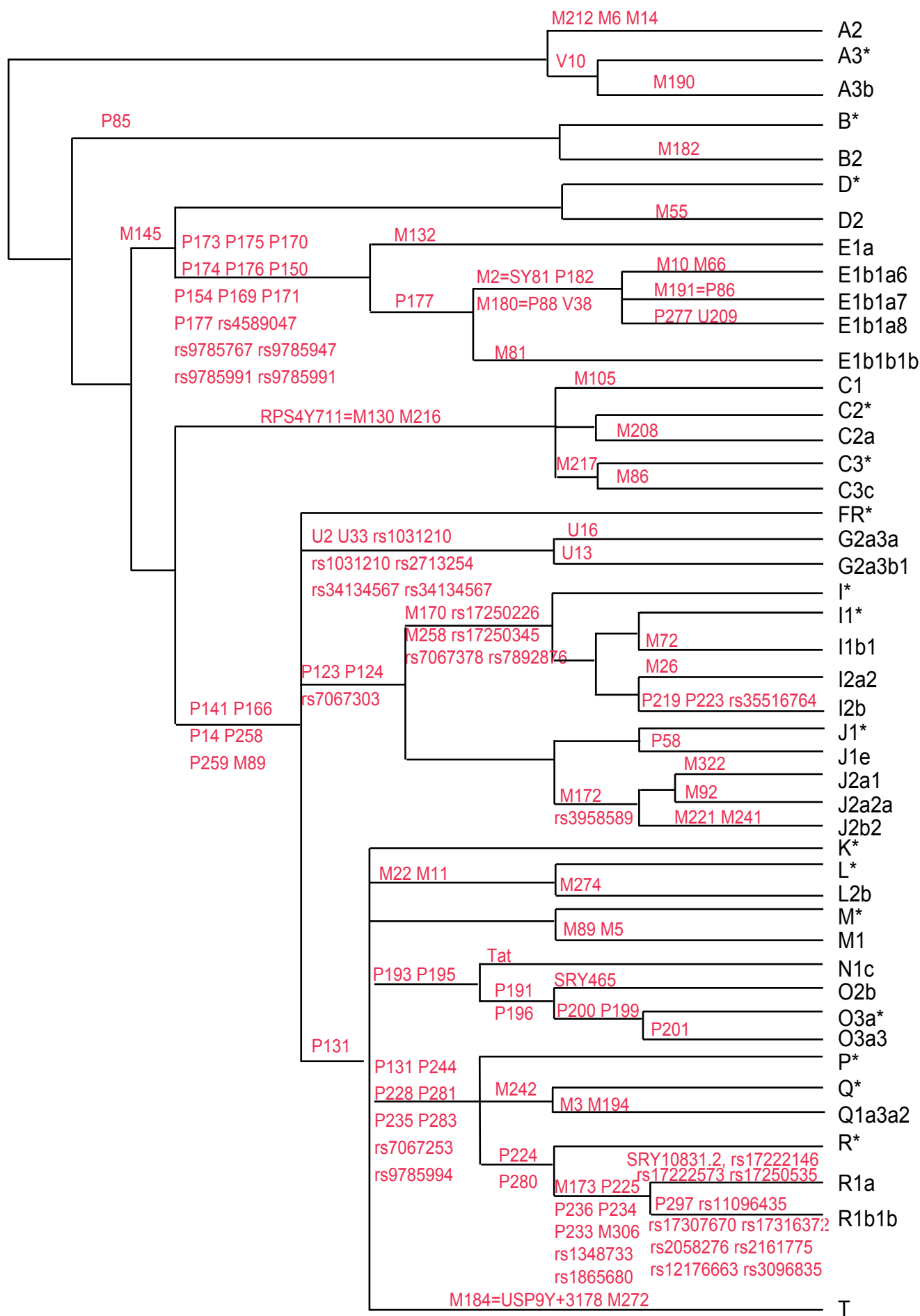

Supplement: Supplementary file 2 — Supplementary material 2 (PDF 133 kb) [file 439_2015_1562_MOESM2_ESM.pdf]
